# Supplementary material for: Active cortical networks promote shunting fast synaptic inhibition in vivo
Source: Neuron. 2023 Nov 15;111(22):3531–3540.e6. doi: 10.1016/j.neuron.2023.08.005 (PMC11913778; doi:10.1016/j.neuron.2023.08.005)
Supplement: Document S1. Figures S1–S7 [file mmc1.pdf]

Neuron, Volume *111*

Supplemental information

Active cortical networks promote

shunting fast synaptic inhibition *in vivo*

Richard J. Burman, Paul J.N. Brodersen, Joseph V. Raimondo, Arjune Sen, and Colin J. Akerman

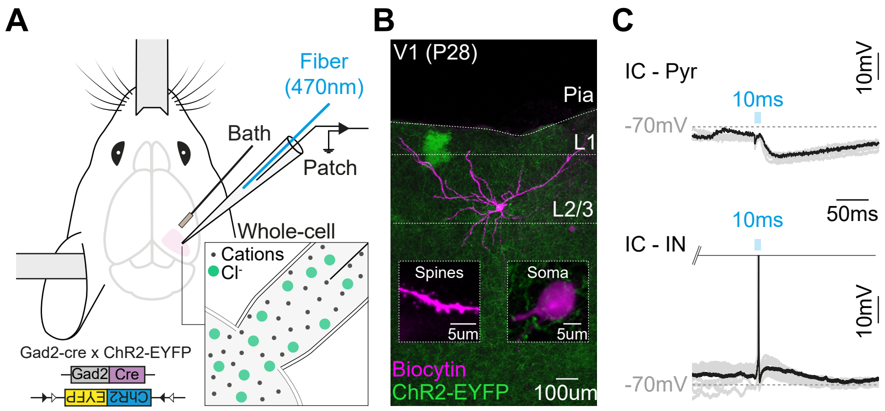

Supplementary Figure 1: Optical activation of Gad2-positive interneurons evokes post-synaptic GABA receptor responses in layer 2/3 cortical pyramidal neurons *in vivo*, related to Figure 1.

(**A**) Schematic of whole-cell patch clamp recording in primary visual cortex (V1) of a transgenic mouse expressing channelrhodopsin-2 fused to enhanced yellow fluorescent protein (ChR2-EYFP) in Gad2-positive interneurons. The Gad2-positive interneurons express Cre-recombinase and are therefore able to remove loxP sites controlling ChR2-YFP expression. Using this system it is possible to selectively express ChR2-YFP only in Gad2-positive GABA-releasing interneurons. To activate ChR2, blue laser light pulses were delivered via a 50 μm optical fibre that was positioned inside and towards the tip of the pipette. This allows for activation of ChR2-expressing pre-synaptic neurons that synapse onto the recorded neuron. Inset shows the whole-cell recording configuration, which allowed biocytin to enter the patched neuron from the internal pipette solution. (**B**) Confocal image of a biocytin-filled layer 2/3 pyramidal neuron stained with streptavidin-fluorophore conjugate, streptavidin-CY3. Inserts show magnified images of the spines and soma of the biocytin-filled neuron. (**C**) Current clamp recordings ('IC') of a light-evoked post-synaptic response (10 ms blue light illumination) from a pyramidal neuron (top, Pyr) and a ChR2-expressing Gad2-positive interneuron (bottom, IN). This meant that it was immediately apparent if we had patched a ChR2-expressing interneuron, as light pulses would elicit action potentials in the recorded neuron.

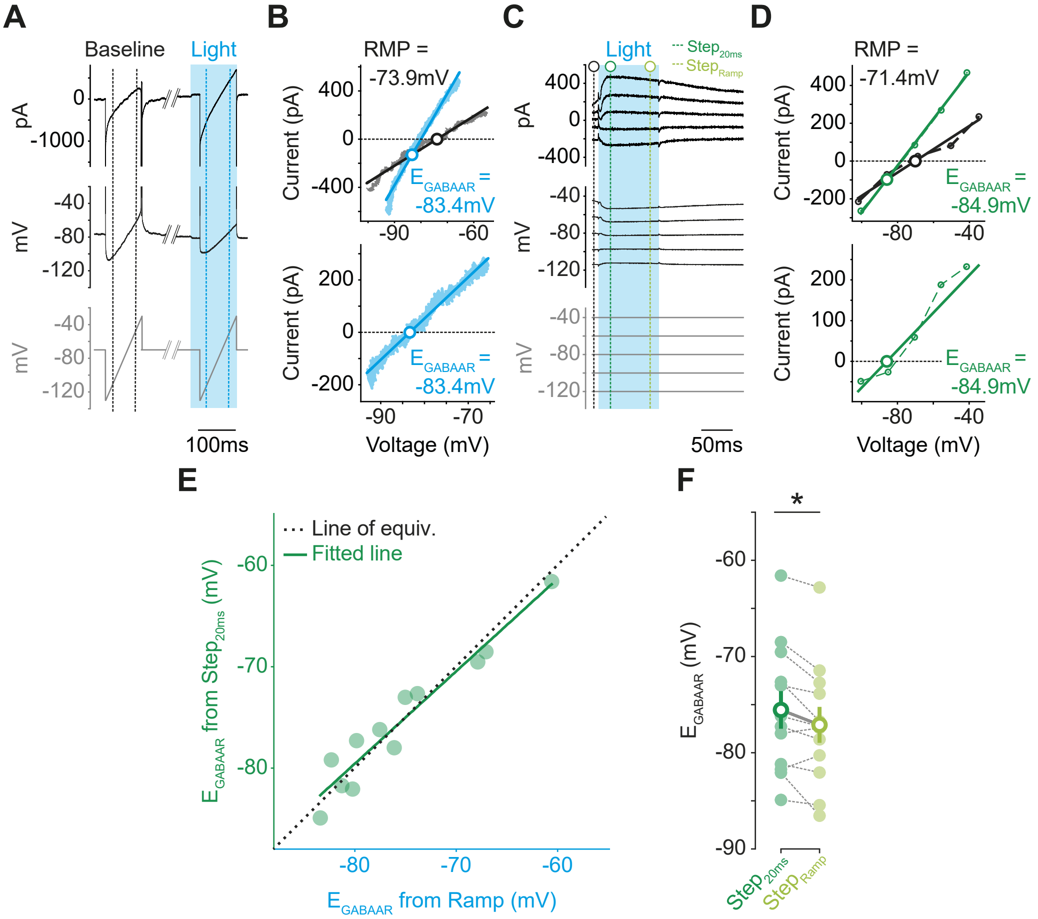

Supplementary Figure 2: *In vivo* voltage ramp protocols provide accurate estimates of synaptic E_{GABAAR}, related to Figure 1.

(**A**) Ramp protocol showing the command holding voltage (bottom, grey), the voltage after applying series resistance correction (R_s = 52.1 MΩ, middle, black), and the current response (black, top). Protocol consisted of a control ramp ('baseline') and a second ramp during which the light-evoked postsynaptic $GABA_A$R-mediated synaptic current was evoked with a blue light pulse ('light'; cyan shaded area). The pairs of vertical dashed lines indicate the regions of the ramps that were analyzed. (**B**) IV plots from a ramp protocol (top) in which the baseline (black) and light (cyan) current responses are shown. The intersection of the two fitted lines represents synaptic E_{GABAAR}, while the point at which the baseline current crosses zero is equivalent to the neuron's RMP. IV plot of the subtracted current (bottom), which corresponds to the synaptic $GABA_A$R current and has a value of zero at E_{GABAAR}. (**C**) A voltage step protocol was used to estimate synaptic E_{GABAAR} in the same neuron as in 'A'. The voltage step protocol (bottom, grey), the voltage after applying series resistance correction (R_s = 50.8 MΩ, middle, black) and current response (top, black) are shown. $GABA_A$R-mediated currents were evoked using light pulses (470 nm, 100 ms, cyan shading). Black vertical dashed line indicates where baseline current was calculated. Dark green vertical dashed line indicates measurement made at the peak of the pure $GABA_A$R-mediated synaptic current, 20 ms from light onset ($Step_{20ms}$).[23] Light green vertical dashed line indicates where measurement was made at the time E_{GABAAR} was estimated from the ramp protocol in the same cell ($Step_{Ramp}$). (**D**) IV plot (top) of the baseline current (black) and the current at $Step_{20ms}$ (dark green). The resulting subtracted current (bottom), corresponds to the synaptic $GABA_A$R current and has a value of zero at E_{GABAAR}. (**E**) A strong correlation was observed between synaptic E_{GABAAR} estimated from the ramp protocol (cyan) and from the voltage step protocol (dark green, $Step_{20ms}$) (*R squared* = 0.92, $p < 0.001$, n = 12 neurons from 6 mice, *Wald test*). (**F**) Population data showing a small (mean amplitude 1.5 mV), but statistically significant difference in E_{GABAAR} measurements between $Step_{20ms}$ and $Step_{Ramp}$ ($Step_{20ms}$: -75.55 ± 1.95 mV vs. $Step_{Ramp}$: -77.10 ± 1.86 mV, p = 0.01, *paired t-test*), consistent with good estimates of E_{GABAAR} during the ramp protocol and a modest potential contribution by $GABA_B$Rs.[1] *, $p < 0.05$.

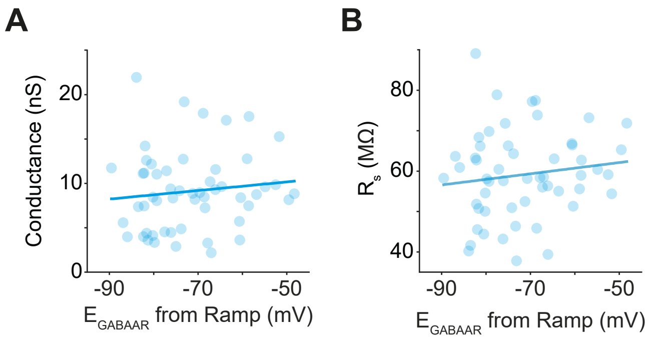

Supplementary Figure 3: Estimates of synaptic E_{GABAAR} are not related to the amplitude of the light-evoked postsynaptic GABA conductance or the series resistance, related to Figure 1.

Related to Figure 1. (**A**) No correlation was observed between synaptic E_{GABAAR} and the conductance of the light-evoked $GABA_AR$ response (*pooled mean* 10.62 ± 0.6 nS, R^2 = 0.01, p = 0.41, n = 54 neurons from 39 mice). Conductance was calculated from the slope of the $GABA_AR$ current recorded during the voltage ramp. (**B**) No correlation was observed between synaptic E_{GABAAR} and the neuron's series resistance (R_s, *pooled mean* 59.13 ± 1.5 MΩ, R^2 = 0.02, p = 0.33).

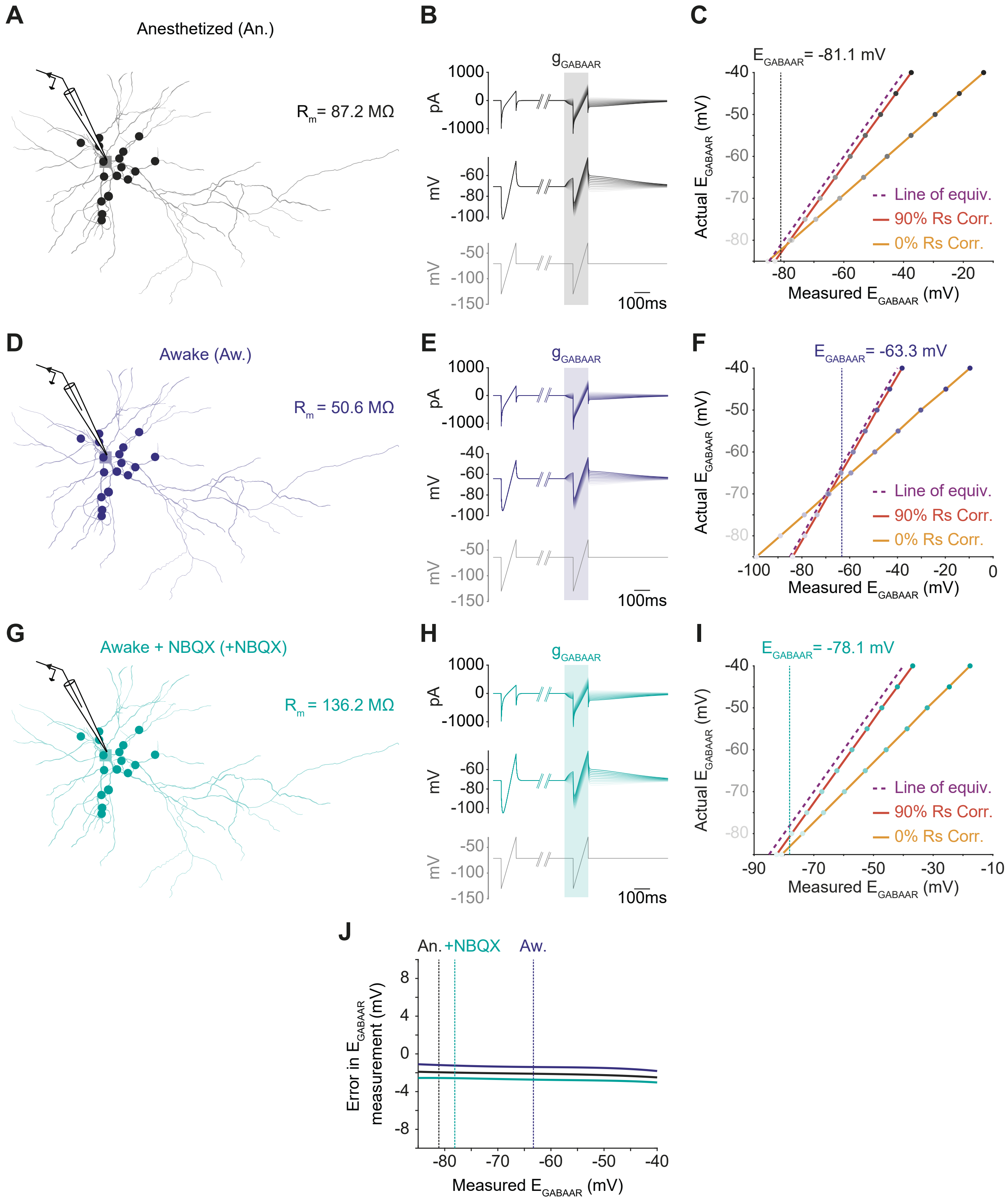

A
Anesthetized (An.)
Rm= 87.2 MΩ
B
gGABAAR
pA
mV
100ms
C
EGABAAR= -81.1 mV
Actual EGABAAR (mV)
Measured EGABAAR (mV)
Line of equiv.
90% Rs Corr.
0% Rs Corr.
D
Awake (Aw.)
Rm = 50.6 MΩ
E
F
EGABAAR= -63.3 mV
G
Awake + NBQX (+NBQX)
Rm = 136.2 MΩ
H
I
EGABAAR= -78.1 mV
J
An. +NBQX Aw.
Error in EGABAAR measurement (mV)
Measured EGABAAR (mV)

Supplementary Figure 4: Differences in intrinsic membrane properties have minimal effects upon estimates of synaptic E_{GABAAR}, related to Figures 2 and 3.

To simulate the effect of intrinsic membrane properties on the estimation of synaptic E_{GABAAR} under different experimental conditions, a multi-compartment model of a L2/3 pyramidal neuron from mouse V1 was constructed using NEURON. (**A**) Morphological reconstruction of the L2/3 pyramidal neuron, with the location of $GABA_A$R-mediated synaptic inputs indicated (filled circles). To recapitulate the anesthetized recording conditions (An., black), the membrane resistance (R_m) in the model neuron was set to the mean experimentally observed value in our recordings from anesthetized cortex. (**B**) Voltage ramp protocols that matched the experimental protocols were applied to the model neuron and used to generate measurements of synaptic E_{GABAAR}, which could be compared to the actual E_{GABAAR} that had been preconfigured in the simulated neuron. The different line intensities indicate simulations using different preconfigured E_{GABAAR} values, ranging from -85 mV (lightest line) to -40 mV (darkest line) in 5 mV increments. (**C**) Plot showing the relationship between the actual E_{GABAAR} and measured E_{GABAAR} in the anesthetized model neuron. The line of equivalence (purple) reflects where the actual and measured E_{GABAAR} values are equal. The relationship between the actual and measured E_{GABAAR} values was determined with zero series resistance correction ('0% R_s corr', orange) and with 90% series resistance correction, which corresponds to experimental conditions ('90% R_s Corr.', red). The vertical dashed line indicates the experimentally observed E_{GABAAR} in the anesthetized state. (**D**) As in 'A', except that to recapitulate the awake state (Aw., blue) the membrane resistance and RMP in the model neuron was set to the mean experimentally observed values in our recordings from awake cortex. (**E**) As in 'B', showing simulations of the awake state. (**F**) As in 'C', with the vertical dashed line indicating the experimentally observed E_{GABAAR} in the awake state. (**G**) As in 'A', except that to recapitulate the awake state under conditions of reduced local network activity (+NBQX, turquoise) the membrane resistance and RMP in the model neuron was set to the mean experimentally observed values in our recordings from awake cortex plus NBQX. (**H**) As in 'B', showing simulations of the awake cortex plus NBQX state. (**I**) As in 'C', with the vertical dashed line indicating the experimentally observed E_{GABAAR} in the awake cortex plus NBQX state. (**J**) The estimated error in E_{GABAAR} measurements (Actual E_{GABAAR} – Measured E_{GABAAR}) plotted for each of the three cortical states (using the 90% R_s corrected data). The experimentally measured E_{GABAAR} for each state is indicated with the vertical dashed lines. Across the three states, the estimated error for measuring synaptic E_{GABAAR} was 1-3 mV.

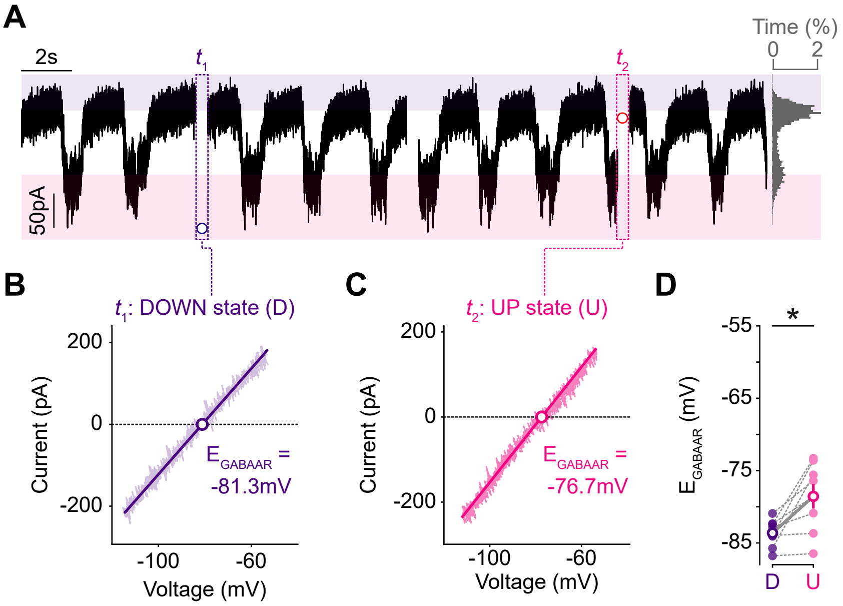

Supplementary Figure 5: Transient fluctuations in E_{GABAAR} are observed across UP and DOWN states in anesthetized cortex, related to Figure 2.

(**A**) More detailed analysis was performed on a subset of anesthetized recordings that exhibited UP and DOWN states. Trace shows a voltage-clamp gramicidin recording from a L2/3 cortical pyramidal neuron in a urethane-anesthetized mouse, showing fluctuations between inward and outward holding currents, corresponding to cortical UP and DOWN states, respectively. Previous work has established that UP states represent periods of high glutamatergic and GABAergic synaptic activity and more depolarized V_m, compared to DOWN states.[2] (**B**) Current-voltage (IV) plot generated from ramp protocol performed during a DOWN state and showing the measured E_{GABAAR}. (**C**) IV plot showing E_{GABAAR} sampled during an UP state. (**D**) Population data showing difference in E_{GABAAR} between cortical DOWN and UP states (DOWN: -83.63 ± 0.68 mV vs. UP: -78.56 ± 1.68 mV, n = 8 cells from 5 mice, p = 0.01, *paired t-test*). This modest synaptic E_{GABAAR} change is consistent with the idea that periods of increased synaptic activity and depolarized V_m favor depolarized synaptic E_{GABAAR} due to an increased Cl^- load.[3,4] *, $p < 0.05$.

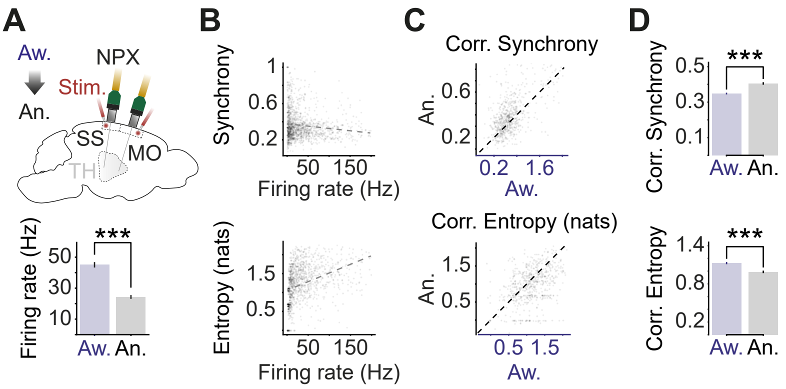

Supplementary Figure 6: Differences in spike rate do not account for differences in synchrony and entropy between the awake and anesthetized cortex, related to Figure 4.

(**A**) Schematic (top) of Neuropixels (NPX) recordings paired with electrical stimulation (Stim.) in somatosensory cortex (SS) and motor cortex (MO) made in the awake (Aw.) and anesthetized (An.) state, from the same mouse. Probes extended from cortex through to thalamus (TH). The firing rate of cortical neurons (bottom; n = 662 neurons from 16 mice) during the awake state was higher than in the anesthetized state (Aw.: 45.1 ± 1.7 Hz vs. An.: 24.3 ± 1.2 Hz, $p < 0.001$, *paired t-test*). (**B**) Scatter plots showing weak relationships between neuron firing rate and synchrony (top; dashed line indicates linear interpolation, $R^2 = 0.02$, $p < 0.001$, *Pearson correlation*) and between firing rate and entropy of the peri-stimulus histogram (bottom; $R^2 = 0.14$, $p < 0.001$, *Pearson correlation*). (**C**) Scatter plots showing synchrony (top) and entropy (bottom) between brain states after correcting for differences in firing rate. (**D**) Population data showing that significant differences in synchrony (Aw.: 0.348 ± 0.005 vs. An.: 0.405 ± 0.007, p <0.001, *paired t-test*) and entropy (Aw.: 1.127 ± 0.017 nats vs. An.: 0.988 ± 0.019 nats, p <0.001, *paired t-test*) remain after correcting for state-dependent differences in firing rate.

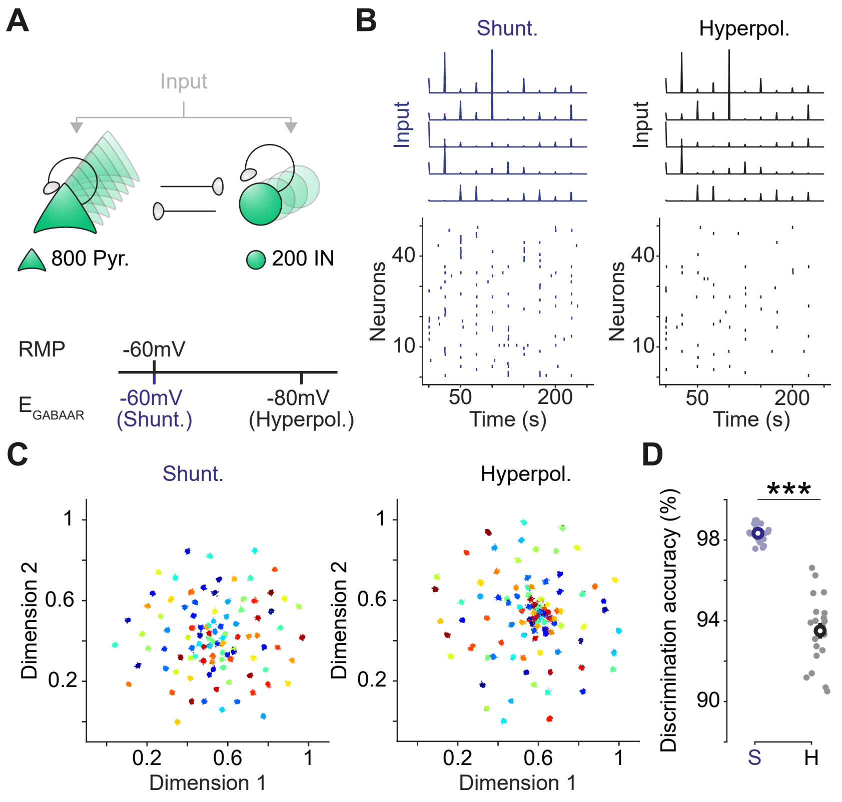

Supplementary Figure 7: Shunting inhibition improves the discriminability of input patterns in neuronal networks, related to Figure 4.

(**A**) Schematic of network model consisting of interconnected excitatory pyramidal neurons (Pyr.) and inhibitory interneurons (IN). E_{GABAAR} in the pyramidal neurons was adjusted relative to the resting membrane potential (RMP) to create two different conditions: a shunting (Shunt.) and a hyperpolarizing (Hyperpol.) E_{GABAAR} condition. For the stimulus inputs (Input), we generated 100 distinct input patterns, each presented 100 times in each condition (shunting or hyperpolarizing E_{GABAAR}). A pattern involved simultaneously delivering a brief current pulse to each neuron, of variable amplitude. (**B**) Example inputs delivered to 5 pyramidal neurons in the model (top). Example raster plots (bottom) show evoked spikes in 50 randomly chosen excitatory neurons in the shunting E_{GABAAR} (left) and hyperpolarizing E_{GABAAR} (right) conditions. (**C**) UMAP projections of the neuronal responses to different input patterns. Each color corresponds to a different input pattern and each dot corresponds to a repeat of that input pattern. In both conditions, responses to the same input pattern form tight clusters. However, shunting E_{GABAAR} results in improved separability of the responses to the different input patterns. (**D**) Discrimination accuracy of k-nearest neighbor classifier trained and tested on input-evoked firing patterns in excitatory neurons (Shunt.: 98.3 ± 0.1 % vs. Hyperpol.: 93.5 ± 0.4 %, n = 20 simulations; $p < 0.001$, *paired t-test*). ***, $p < 0.001$.

Supplementary References:

1. Davies, C.H., Davies, S.N., and Collingridge, G.L. (1990). Paired-pulse depression of monosynaptic GABA-mediated inhibitory postsynaptic responses in rat hippocampus. The Journal of Physiology *424*, 513–531. 10.1113/jphysiol.1990.sp018080.

2. Haider, B., Duque, A., Hasenstaub, A.R., and McCormick, D.A. (2006). Neocortical Network Activity In Vivo Is Generated through a Dynamic Balance of Excitation and Inhibition. Journal of Neuroscience *26*, 4535–4545. 10.1523/JNEUROSCI.5297-05.2006.

3. Doyon, N., Vinay, L., Prescott, S.A., and De Koninck, Y. (2016). Chloride Regulation: A Dynamic Equilibrium Crucial for Synaptic Inhibition. Neuron *89*, 1157–1172. 10.1016/j.neuron.2016.02.030.

4. Staley, K., Soldo, B., and Proctor, W. (1995). Ionic mechanisms of neuronal excitation by inhibitory GABAA receptors. Science *269*, 977–981. 10.1126/science.7638623.
