# Supplementary material for: Active cortical networks promote shunting fast synaptic inhibition in vivo
Source: Neuron. 2023 Nov 15;111(22):3531–3540.e6. doi: 10.1016/j.neuron.2023.08.005 (PMC11913778; doi:10.1016/j.neuron.2023.08.005)
Supplement: Document S2. Article plus supplemental information [file mmc2.pdf]

Neuron

Report

Active cortical networks promote shunting fast synaptic inhibition *in vivo*

Graphical abstract

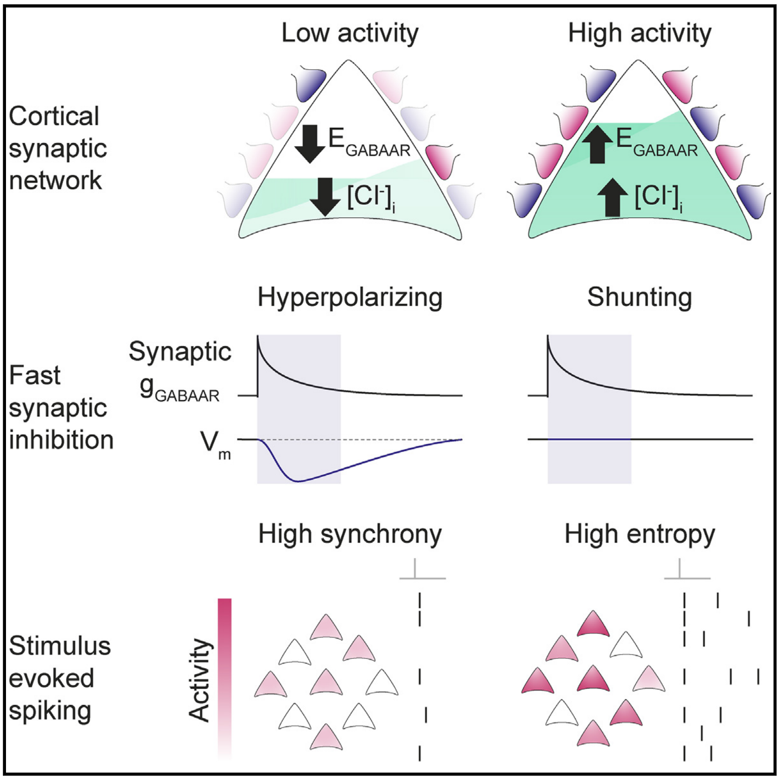

Authors

Richard J. Burman,
Paul J.N. Brodersen,
Joseph V. Raimondo, Arjune Sen,
Colin J. Akerman

Correspondence

colin.akerman@pharm.ox.ac.uk

In brief

In this study, Burman et al. demonstrate that the way in which fast synaptic inhibition operates in the intact mammalian cortex is linked to the level of activity within the network. The authors further show that this dynamic inhibition has profound implications for how the cortex responds to incoming stimuli.

Highlights

- *In vivo* gramicidin recordings reveal the driving force for synaptic $GABA_ARs$
- Synaptic E_{GABAAR} favors shunting inhibition in the awake cortex
- The high levels of cortical activity raise E_{GABAAR} to promote shunting inhibition
- Shunting inhibition promotes local network desynchronization and response flexibility

Burman et al., 2023, Neuron *111*, 3531–3540
November 15, 2023 © 2023 The Author(s). Published by Elsevier Inc.
https://doi.org/10.1016/j.neuron.2023.08.005

Neuron

CellPress
OPEN ACCESS

Report

Active cortical networks promote shunting fast synaptic inhibition *in vivo*

Richard J. Burman,[1,2,4] Paul J.N. Brodersen,[1,4] Joseph V. Raimondo,[3] Arjune Sen,[2] and Colin J. Akerman[1,5,*]
[1]Department of Pharmacology, University of Oxford, Oxford, OX1 3QT, UK
[2]Oxford Epilepsy Research Group, Nuffield Department of Clinical Neurosciences, University of Oxford, Oxford, OX3 9DU, UK
[3]Division of Cell Biology, Department of Human Biology, Neuroscience Institute and Institute of Infectious Diseases and Molecular Medicine, University of Cape Town, Cape Town, 7935, South Africa
[4]These authors contributed equally
[5]Lead contact
*Correspondence: colin.akerman@pharm.ox.ac.uk
https://doi.org/10.1016/j.neuron.2023.08.005

SUMMARY

Fast synaptic inhibition determines neuronal response properties in the mammalian brain and is mediated by chloride-permeable ionotropic GABA-A receptors ($GABA_A$Rs). Despite their fundamental role, it is still not known how $GABA_A$Rs signal in the intact brain. Here, we use *in vivo* gramicidin recordings to investigate synaptic $GABA_A$R signaling in mouse cortical pyramidal neurons under conditions that preserve native transmembrane chloride gradients. In anesthetized cortex, synaptic $GABA_A$Rs exert classic hyperpolarizing effects. In contrast, $GABA_A$R-mediated synaptic signaling in awake cortex is found to be predominantly shunting. This is due to more depolarized $GABA_A$R equilibrium potentials (E_{GABAAR}), which are shown to result from the high levels of synaptic activity that characterize awake cortical networks. Synaptic E_{GABAAR} observed in awake cortex facilitates the desynchronizing effects of inhibitory inputs upon local networks, which increases the flexibility of spiking responses to external inputs. Our findings therefore suggest that $GABA_A$R signaling adapts to optimize cortical functions.

INTRODUCTION

Synaptic inhibition is tightly coupled to synaptic excitation and plays a key role in cortical computations,[1] including modulating sensory response properties[2,3] and oscillatory activities.[4] Fast synaptic inhibition in cortex is mediated by ionotropic GABA-A receptors ($GABA_A$Rs), which are mainly permeable to chloride (Cl^-).[5–7] The inhibitory effects that $GABA_A$Rs have upon a neuron therefore depend upon the local transmembrane Cl^- gradient, which reflects a dynamic equilibrium between Cl^- extrusion and intrusion processes.[8–10]

When the $GABA_A$R equilibrium potential (E_{GABAAR}) is more negative than the membrane potential (V_m), $GABA_A$R activation will lead to membrane hyperpolarization. If E_{GABAAR} is close to V_m, $GABA_A$R activation would have minimal effects upon V_m, and the inhibitory effects would be primarily mediated by local effects on the membrane resistance (R_m)—a phenomenon known as "shunting inhibition."[11,12] These different forms of signaling determine how inhibition is temporally integrated with incoming excitatory synaptic inputs.[13,14] Thus, appreciating how fast synaptic inhibition operates is fundamental to understanding how neuronal and network activity are regulated in the intact brain.

Previous work has identified the contribution of different transmembrane Cl^- fluxes in determining synaptic E_{GABAAR} in cortex. This includes Cl^- effluxes mediated by the potassium-chloride co-transporter (KCC2)[15,16] as well as Cl^- influxes such as those mediated by $GABA_A$Rs themselves, which can vary depending upon a neuron's activity.[17,18] Previous investigations of synaptic E_{GABAAR} relied heavily upon *in vitro* investigation and were therefore influenced by the distorted fluxes that operate under these recording conditions.[9] Consequently, there is a lack of evidence regarding how fast synaptic inhibition operates in the intact cortex.

To address this gap, we combine optogenetic activation of GABAergic synaptic inputs with *in vivo* gramicidin perforated patch-clamp recordings. This enables us to measure synaptic E_{GABAAR} and $GABA_A$R driving forces in the intact rodent cortex, under conditions in which transmembrane Cl^- gradients are preserved. We demonstrate that, in contrast to the anesthetized cortex, pyramidal neurons of the awake cortex exhibit a relatively high synaptic E_{GABAAR}, which is close to resting V_m and generates a clear preference for shunting fast synaptic inhibition. This depolarized E_{GABAAR} results from the high levels of synaptic activity in the awake cortex, and the resulting shunting synaptic inhibition is well placed to reduce synchrony within local cortical networks and increase the flexibility of responses to external input.

This is an open access article under the CC BY license (http://creativecommons.org/licenses/by/4.0/).

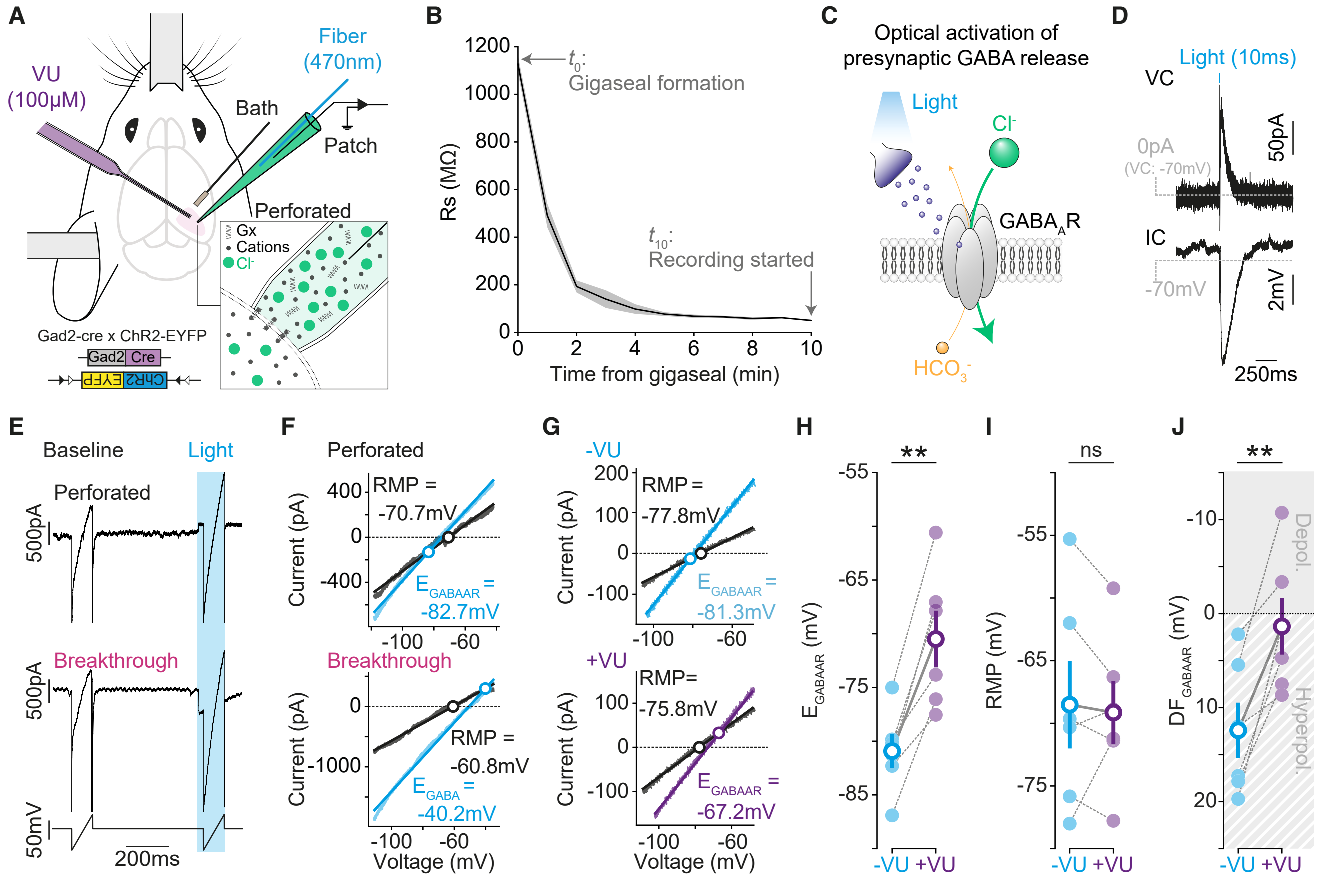

Figure 1. Measuring synaptic E_{GABAAR} and synaptic $GABA_AR$ driving forces *in vivo*

(A) Setup for performing gramicidin perforated patch-clamp recordings in V1, in combination with optogenetic activation of local GABAergic synaptic inputs.

(B) Series resistance (R_s) over time, shown for neurons that met the criteria for successful perforation (mean ± SEM; n = 10 neurons, 7 mice).

(C) Optogenetic approach elicits presynaptic GABA release and activates postsynaptic $GABA_AR$s, which are mainly permeable to Cl^- and, to a lesser extent, bicarbonate (HCO_3^-).[6]

(D) Light-evoked currents (voltage-clamp, VC) and potentials (current-clamp, IC).

(E) Ramp protocol in perforated configuration (top) and following breakthrough into whole-cell configuration (middle). The voltage protocol before R_s correction is also shown (bottom) and consisted of a control ramp ("baseline") and a second ramp during the light-evoked synaptic GABA conductance ("light").

(F) IV plots for baseline (black) and light (cyan) ramps performed under perforated (top) and breakthrough (bottom) conditions. The reversal potential of the baseline current ("resting membrane potential [RMP]") and E_{GABAAR} are indicated with circles.

(G) IV plots showing synaptic E_{GABAAR} before (−VU, cyan, top) and after VU application (+VU, purple, bottom).

(H) Population data (n = 6 neurons from 6 mice) showed a depolarizing shift in synaptic E_{GABAAR} after VU (−VU: −80.9 ± 1.6 mV vs. +VU: −70.9 ± 2.6 mV; p = 0.002, paired t test).

(I) VU did not affect RMP (−VU: −68.5 ± 3.5 mV vs. +VU: −69.1 ± 2.5 mV; p = 0.73, paired t test).

(J) VU caused a depolarizing shift in $GABA_AR$ driving force (DF_{GABAAR} = RMP − E_{GABAAR}, −VU: 12.4 ± 2.9 mV vs. +VU: 1.4 ± 3.0 mV; p = 0.006, paired t test). ns, not significant; **p < 0.01.

RESULTS

Measuring the equilibrium potential and driving force for synaptic $GABA_A$Rs *in vivo*

To study $GABA_A$R-mediated synaptic signaling *in vivo*, we established gramicidin perforated patch-clamp recordings from layer 2/3 (L2/3) pyramidal neurons in primary visual cortex (V1) of urethane-anesthetized mice aged between six and eight weeks (Figures 1A and S1). Similar to *in vitro* findings,[19,20] the perforation of the neuronal membrane with cation-selective gramicidin pores was marked by a decrease in the series resistance (R_s; Figure 1B). During the 10 min following gigaseal formation we observed a 20-fold decrease in R_s (mean $R_{s\text{-}0\ min}$: 1,126.9 ± 35.8 MΩ vs. $R_{s\text{-}10\ min}$: 50.2 ± 3.8 MΩ), which provided stable conditions for studying synaptic responses. By using transgenic mice that express channelrhodopsin-2 (ChR2) in Gad2-positive neurons (Figures 1A and 1C), we were able to initiate presynaptic GABA release by optically activating the axons of nearby GABAergic interneurons.[21,22] This resulted in postsynaptic $GABA_A$R responses in the L2/3 pyramidal neuron, which could be recorded in either current or voltage clamp (Figures 1D and S1C).

To measure synaptic E_{GABAAR} *in vivo*, we combined our optogenetic approach with voltage ramp protocols (Figure 1E) to minimize disruption to transmembrane Cl^- gradients and generate current-voltage (IV) plots from which the resting V_m (RMP) and E_{GABAAR} can be determined (Figure 1F). The internal pipette solution contained high Cl^- (~150 mM) so that the integrity of the patch could be confirmed at the end of a recording by electing to break through into whole-cell mode (Figures 1E and 1F). To check that our voltage ramp protocols provided an accurate estimate of synaptic E_{GABAAR}, results were compared to voltage step protocols performed in the same neuron (Figure S2). Step protocols take longer to perform but offer the chance to analyze synaptic E_{GABAAR} at a defined time following presynaptic GABA release, thereby further isolating the $GABA_AR$ response (Figure S2).[23] The estimates of synaptic E_{GABAAR} from ramp protocols were equivalent to those made using step protocols (Figure S2E), and measurements from the ramp protocols were not related to the amplitude of the synaptic $GABA_AR$ response or the neuron's R_s (Figure S3). To further validate the setup, we assessed sensitivity to changes in synaptic E_{GABAAR} caused by altering the balance of transmembrane Cl^- fluxes. As expected, application of the selective KCC2 antagonist, VU0463271 (VU), resulted in a depolarizing shift in synaptic E_{GABAAR} (Figures 1G and 1H), consistent with the blocking of a Cl^- efflux. While VU caused a depolarizing shift in E_{GABAAR}, there was no detectable change in RMP (Figure 1I), and consequently, there was a depolarizing shift in the driving force for $GABA_AR$s (DF_{GABAAR} = RMP − E_{GABAAR}; Figure 1J).

Awake cortex exhibits more depolarized synaptic E_{GABAAR} and shunting fast synaptic inhibition

We next investigated synaptic $GABA_AR$ signaling in the awake cortex (Figure 2A). In keeping with previous whole-cell studies,[24,25] our gramicidin recordings in head-fixed mice revealed that L2/3 pyramidal neurons in the awake cortex exhibit high levels of synaptic activity. Compared with anesthetized cortex, awake neurons exhibited more depolarized V_m (Figures 2B and 2C) and larger fluctuations in their subthreshold V_m (Figure 2D). This is consistent with the elevated levels of excitatory and inhibitory synaptic activity reported in the awake cortex.[24,25] The R_m was also lower in the awake recordings (An.: 87.2 ± 6.1 MΩ, n = 14 neurons, 8 mice vs. Aw.: 50.6 ± 6.4 MΩ, n = 13 neurons, 10 mice, $p < 0.0001$, unpaired t test), consistent with the increased membrane conductance associated with high levels of synaptic activity.[26] The membrane properties we observed in the anesthetized state were consistent with what has been reported with other anesthetics,[3,25,26] suggesting that a more hyperpolarized mean V_m and lower levels of synaptic activity are a common feature of anesthetized cortex.

The high level of synaptic activity in awake cortex includes strong activation of $GABA_AR$-containing synapses.[3] This generates conditions that are predicted to increase the Cl^- influxes (i.e., a "Cl^- load") experienced by a cortical neuron,[9,27] particularly as more depolarized V_m values will increase the driving force for Cl^- to enter via the $GABA_AR$ (Figure 2E). Indeed, strong $GABA_AR$ activation can alter intracellular Cl^- levels and affect E_{GABAAR} via changes in the equilibrium potential of Cl^- (E_{Cl}),[9,27] while the equilibrium potential for bicarbonate is more stable owing to buffering mechanisms.[28] We hypothesized that the conditions of the awake cortex would impact transmembrane Cl^- gradients and thereby affect synaptic $GABA_AR$ signaling. To test this prediction, we used our optogenetic approach to compare synaptic $GABA_AR$ responses between the anesthetized cortex and awake cortex. First, current clamp recordings revealed that synaptic $GABA_AR$ responses in the awake cortex produced V_m deflections that remained close to the RMP and could be depolarizing or hyperpolarizing (Figure 2F). This indicated a net shunting effect for synaptic $GABA_AR$ in the awake cortex, consistent with a different DF_{GABAAR} that could be caused by a more depolarized synaptic E_{GABAAR}. We confirmed this by performing ramp protocols in voltage clamp, demonstrating that synaptic E_{GABAAR} was more depolarized in the awake cortex (Figures 2G and 2H). While the RMP was also more depolarized in the awake cortex (Figure 2I), the net effect was to move the synaptic DF_{GABAAR} in a depolarized direction when compared with the anesthetized cortex (Figure 2J). Importantly, the synaptic DF_{GABAAR} in the awake cortex was not different from zero, consistent with the conclusion that $GABA_AR$-mediated synaptic signaling favors shunting inhibitory effects in the awake state. These differences were not related to how the synaptic $GABA_AR$s were activated because the amplitude of the $GABA_AR$ conductances were comparable in the anesthetized cortex and awake cortex (Figure 2K). Meanwhile, modeling of synaptic $GABA_AR$ responses in biologically realistic neurons confirmed that differences in synaptic E_{GABAAR} could not be explained by the intrinsic membrane properties in anesthetized and awake cortices (Figure S4). Finally, more detailed analysis revealed modest synaptic E_{GABAAR} changes within anesthetized recordings, which were consistent with the general principle[9,27] that increased synaptic activity and depolarized V_m result in more depolarized synaptic E_{GABAAR} because of an increased Cl^- load (Figure S5).

Network activity in awake cortex raises synaptic E_{GABAAR} and promotes shunting fast synaptic inhibition

Having established that awake cortex favors shunting synaptic $GABA_AR$ responses, we explored whether the more depolarized synaptic E_{GABAAR} is caused by the high levels of synaptic activity that exist in the awake state. To test this, we examined the effects of reducing local network activity in awake cortex by blocking glutamatergic signaling with a local injection of the AMPA receptor antagonist, NBQX (Figure 3A). Following NBQX injection, the distribution and mean V_m of cortical neurons were more hyperpolarized (Figures 3B and 3C), and fluctuations in subthreshold V_m were greatly decreased (Figure 3D), consistent with the effective suppression of synaptic activity to the recorded neuron. The reduction in local network activity was also reflected in the neuron's R_m, which was higher in NBQX, again consistent with decreased synaptic activity (Aw.: 50.6 ± 6.4 MΩ, n = 13 neurons, 10 mice vs. +NBQX: 136.2 ± 11.9 MΩ, n = 10 neurons, 7 mice, $p < 0.0001$, unpaired t test).

To investigate whether the levels of local network activity determine the nature of GABAergic signaling in the awake cortex, the polarity of synaptic $GABA_AR$ responses was compared across recordings performed with and without

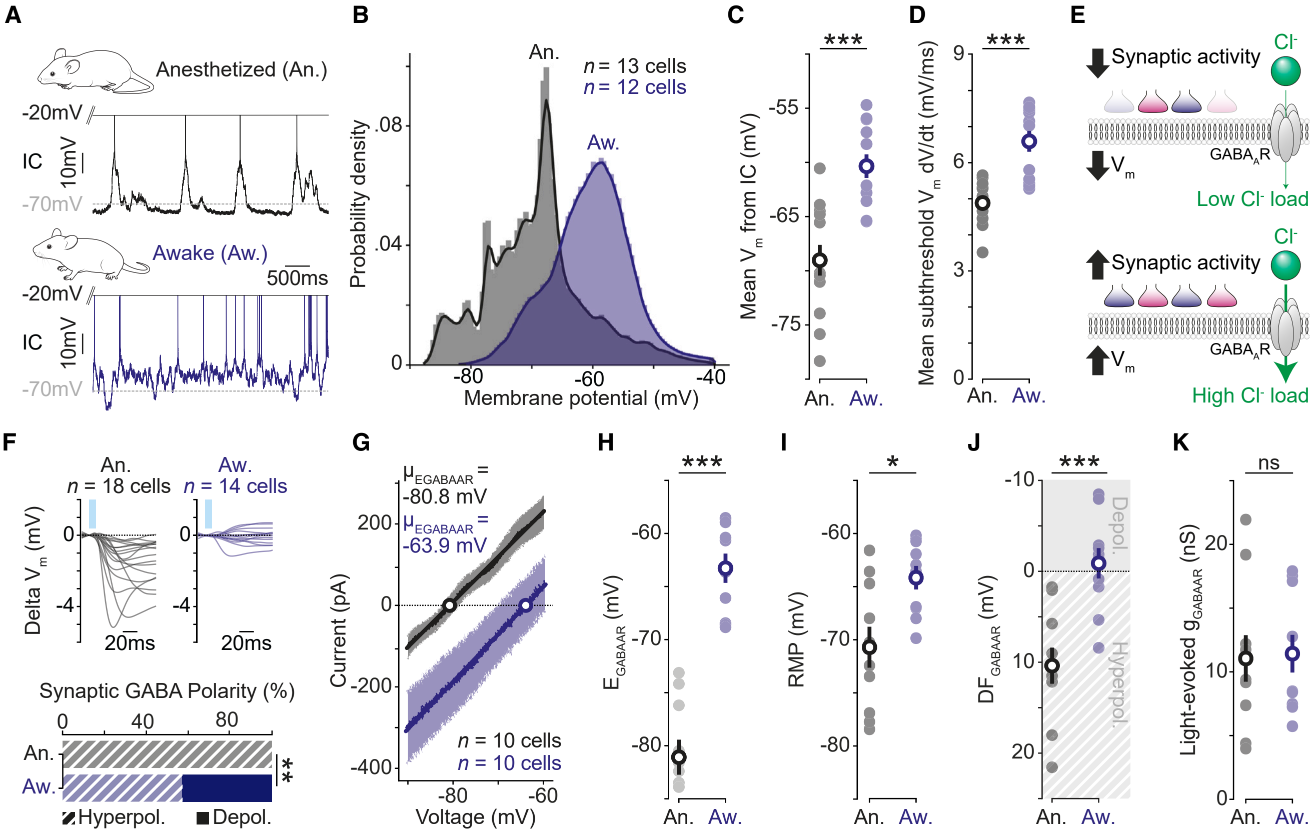

Figure 2. Awake cortex exhibits depolarized synaptic E_{GABAAR} and shunting fast synaptic inhibition

(A) Current clamp (IC) recording of spontaneous activity from a L2/3 pyramidal neuron in an anesthetized (An., black, top) and awake mouse (Aw., blue, bottom).

(B) Probability density function for V_m in anesthetized (n = 13 cells, 7 mice) and awake (n = 12 cells, 10 mice) cortex. Awake data are also used in Figure 3.

(C) Mean V_m was more depolarized in awake cortex (An.: −69.1 ± 1.4 mV vs. Aw.: −60.3 ± 1.1 mV; $p < 0.001$, one-way ANOVA with Bonferroni correction).

(D) Mean change in subthreshold V_m was greater in awake cortex (An.: 4.9 ± 0.2 mV/ms vs. Aw.: 6.6 ± 0.3 mV/ms; $p < 0.001$, one-way ANOVA with Bonferroni correction).

(E) Illustration of how a more depolarized V_m and higher synaptic activity are conducive to greater $GABA_AR$-mediated Cl^- influxes.

(F) Averaged light-evoked postsynaptic IC responses in anesthetized (*n* = 18 cells, 10 mice) and awake (n = 14 cells, 11 mice) cortex. Responses in awake cortex produced V_m deflections that remained close to the RMP and could be depolarizing or hyperpolarizing (An.: Depol. 0/18 vs. Aw.: Depol. 6/14; p = 0.003, Fisher-Exact test).

(G) Summary IV plot of all anesthetized (n = 10 cells, 6 mice) and awake (n = 10 cells, 9 mice) light-evoked GABA currents reveal a more depolarized synaptic E_{GABAAR} in awake cortex.

(H) Synaptic E_{GABAAR} is more depolarized in awake cortex (An.: −81.1 ± 1.7 mV vs. Aw.: −63.3 ± 1.4 mV; $p < 0.001$, one-way ANOVA with Bonferroni correction).

(I) Voltage-clamp recordings confirmed a more depolarized RMP in awake cortex (An.: −70.7 ± 1.9 mV vs. Aw.: −64.2 ± 1.1 mV; p = 0.02, one-way ANOVA with Bonferroni correction).

(J) $GABA_AR$ driving force (DF_{GABAAR} = RMP − E_{GABAAR}) was more depolarized in awake cortex (An.: 10.4 ± 2.0 mV vs. Aw.: −0.9 ± 1.7 mV; $p < 0.001$, one-way ANOVA with Bonferroni correction). Awake DF_{GABAAR} was not different to zero, consistent with synaptic $GABA_A$Rs exerting a predominantly shunting effect (An.: p = 0.0006, one-sample t test; Aw.; p = 0.61, one-sample t test).

(K) Light-evoked synaptic GABA conductances did not differ between the anesthetized and awake cortex (An. 11.04 ± 1.8 nS: vs. Aw.: 11.4 ± 1.5 nS, p = 0.92, one-way ANOVA with Bonferroni correction). ns, not significant; *$p < 0.05$; **$p < 0.01$; ***$p < 0.001$.

NBQX (Figure 3E). In the quietened network state, light-evoked responses recorded in current clamp were found to be exclusively hyperpolarizing, which differed from the active awake state (Figure 3E). Consistent with this, reducing local network activity caused a hyperpolarizing shift in synaptic E_{GABAAR} (Figures 3F and 3G). When combined with the negative shift in RMP (Figure 3H), the net effect of reducing local network activity was to cause a hyperpolarizing shift in DF_{GABAAR}, such that the synaptic DF_{GABAAR} was now strongly hyperpolarizing (Figure 3I). Importantly, the effects of reducing local network activity with NBQX were not related to effects on the optogenetic paradigm itself, as the amplitude of the light-evoked synaptic $GABA_AR$ conductances were unaffected by the AMPA receptor antagonist (Figure 3J). More generally, this confirmed that the measurements of synaptic E_{GABAAR} were not contaminated by glutamatergic conductances. Also, the differences in synaptic E_{GABAAR} could not be explained by the neurons' intrinsic membrane properties (Figure S4).

CellPress
OPEN ACCESS

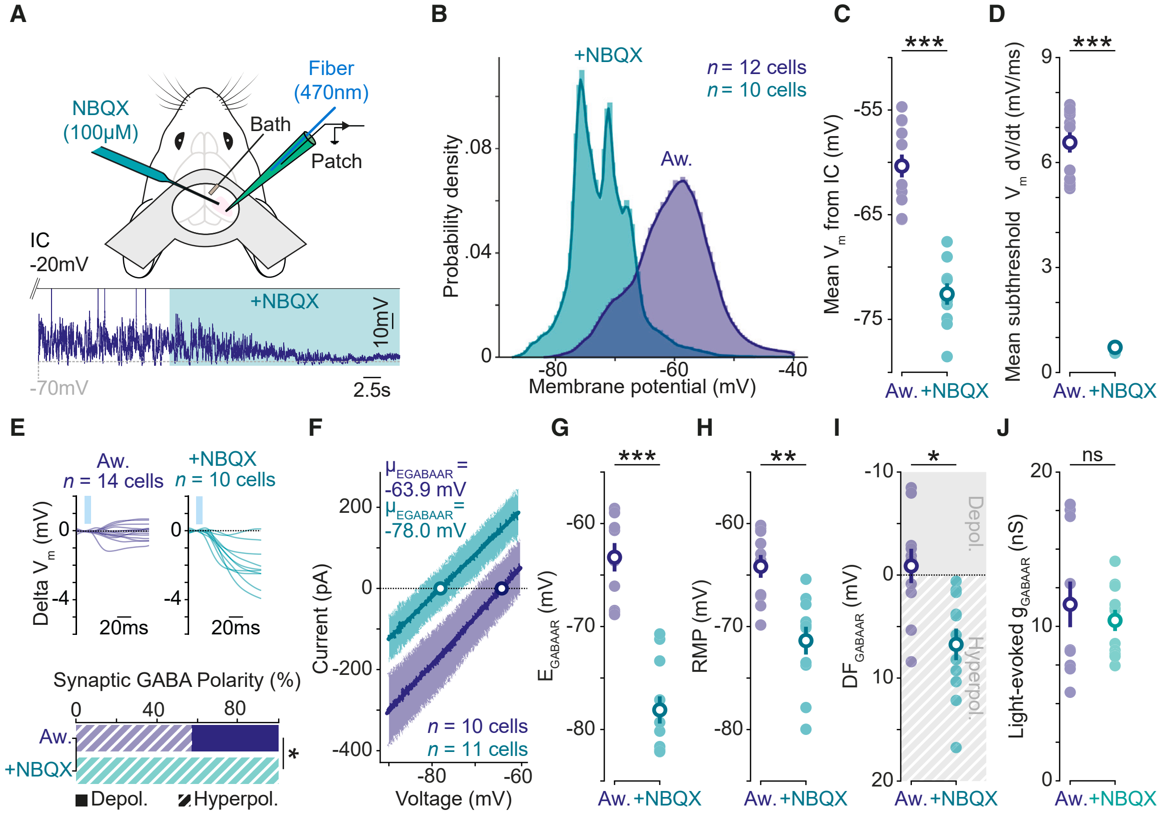

Figure 3. High network activity raises synaptic E_{GABAAR} and promotes shunting fast synaptic inhibition in awake cortex

(A) Setup (top) and current-clamp (IC) recording (bottom) showing the effects of reducing local network activity by acute, local delivery of NBQX (100 μM) in awake cortex.

(B) Probability density function for V_m in control awake cortex and following NBQX (Aw. blue: n = 12 neurons, 10 mice vs. +NBQX, turquoise: n = 10 neurons, 7 mice). Control data from awake group presented in Figure 2.

(C) Reducing local network activity caused a hyperpolarizing shift in mean V_m (Aw.: −60.3 ± 1.1 mV vs. +NBQX: −72.6 ± 1.0 mV; $p < 0.001$, one-way ANOVA with Bonferroni correction).

(D) Reducing local network activity caused a decrease in the mean change in subthreshold V_m (Aw: 6.6 ± 0.3 mV/ms vs. +NBQX: 0.7 ± 0.1 mV/ms; $p < 0.001$, one-way ANOVA with Bonferroni correction).

(E) Averaged light-evoked postsynaptic IC responses (top; Aw., blue: n = 14 neurons, 11 mice vs. +NBQX, turquoise: n = 10 neurons, 7 mice). Reducing local network activity caused a hyperpolarizing shift in the polarity of light-evoked GABA currents (bottom; Aw.: Depol. 6/14 vs. +NBQX: Depol. 0/10; p = 0.03, Fisher-Exact test).

(F) Summary IV plots of light-evoked GABA currents reveal more hyperpolarized synaptic E_{GABAAR} values following local NBQX (Aw.: n = 10 neurons, 9 mice vs. +NBQX: n = 11 neurons, 7 mice).

(G) Reducing local network activity led to more hyperpolarized synaptic E_{GABAAR} (Aw: −63.3 ± 1.4 mV vs. +NBQX: −78.1 ± 1.3 mV; $p < 0.001$, one-way ANOVA with Bonferroni correction).

(H) Reducing local network activity caused a hyperpolarizing shift in RMP (Aw.: −64.2 ± 1.1 mV vs. +NBQX: −71.4 ± 1.4 mV; p = 0.009, one-way ANOVA with Bonferroni correction).

(I) Reducing local network activity caused a hyperpolarizing shift in DF_{GABAAR} (Aw.: −0.9 ± 1.7 mV vs. +NBQX: 6.7 ± 1.5 mV; p = 0.03, one-way ANOVA with Bonferroni correction). DF_{GABAAR} became different from zero (Aw.: p = 0.61, one-sample t test; +NBQX: p = 0.001, one-sample t test).

(J) Reducing local network activity did not affect light-evoked synaptic GABA conductances (Aw.: 11.4 ± 1.5 nS vs. +NBQX: 10.4 ± 0.7 nS; p = 0.78, one-way ANOVA with Bonferroni correction). ns, not significant; *$p < 0.05$; **$p < 0.01$; ***$p < 0.001$.

Shunting inhibition promotes local network desynchronization and response flexibility

The nature of synaptic inhibition affects how neurons integrate incoming excitatory input.[12,13] To explore the implications of the shunting synaptic E_{GABAAR} observed in the awake state, we first analyzed a publicly available data set of high-density Neuropixels (NPXs) recordings from regular spiking neurons in mouse cortex.[29,30] These data allowed us to compare how the same population of neurons respond to a stimulus under awake and isoflurane-anesthetized conditions (Figure 4A). In the awake state, electrical stimuli delivered to cortex evoked spiking activity, with neurons exhibiting variability in terms of when they spiked relative to one another. In contrast, in the anesthetized state, the same stimuli evoked less overall activity, and spiking

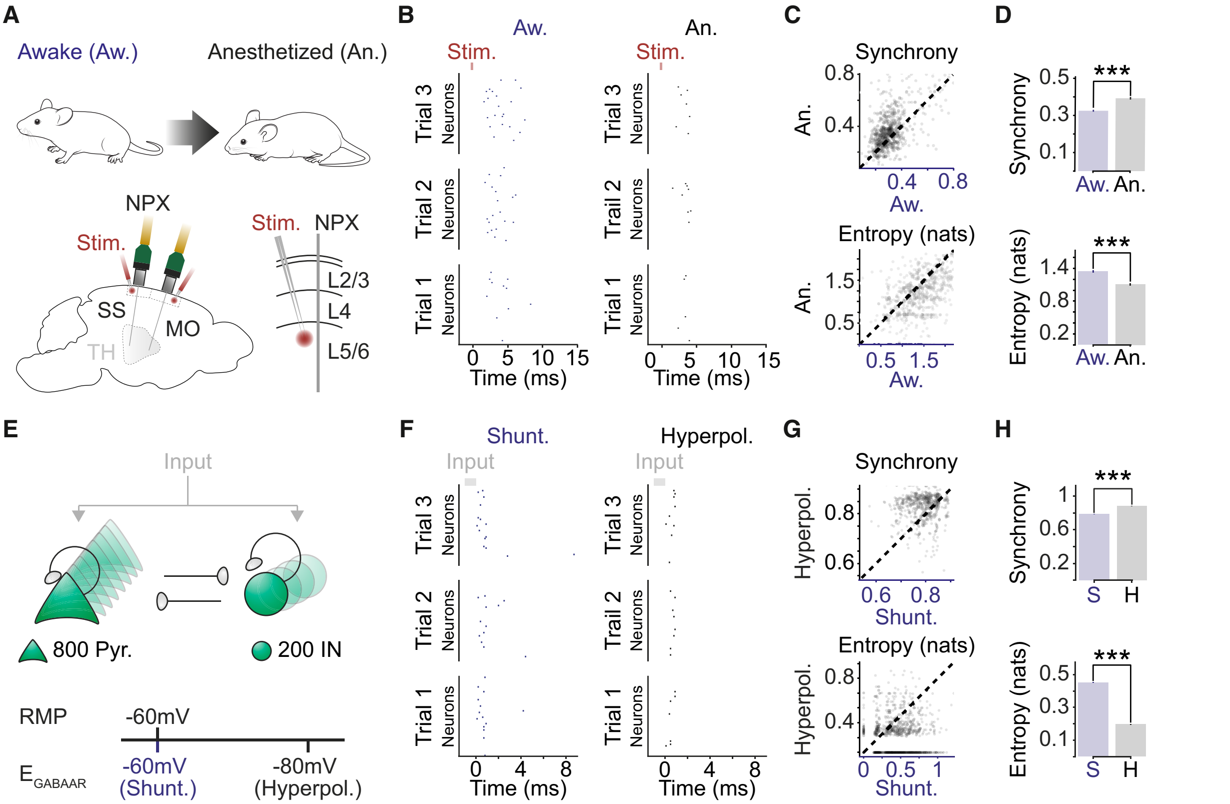

Figure 4. Shunting inhibition promotes local network desynchronization and response flexibility

(A) High-density Neuropixels (NPXs) recordings were used to compare spiking activity in the same cortical neurons under awake and anesthetized conditions.

(B) Example raster plots show the spiking activity of a population of neurons in somatosensory cortex (SS). Spiking activity is shown across three stimulation trials for each condition.

(C) Neuronal synchrony on a trial-to-trial basis (top) and the entropy of the peri-stimulus histogram (bottom) were calculated for each of the 662 recorded neurons under awake and anesthetized conditions (17 probe recordings in 16 mice). Each dot corresponds to a single neuron and the dashed line indicates the line of equality.

(D) Neurons in the awake condition exhibited lower synchrony (top; Aw.: 0.325 ± 0.005 vs. An.: 0.393 ± 0.007, $p < 0.001$, paired t test) and higher entropy (bottom; Aw.: 1.347 ± 0.018 nats vs. An.: 1.111 ± 0.020 nats, $p < 0.001$, paired t test).

(E) Schematic of network model consisting of interconnected excitatory pyramidal neurons (Pyr.) and inhibitory interneurons (IN). E_{GABAAR} in the pyramidal neurons was adjusted relative to the RMP to create two different conditions: a shunting (Shunt.) and a hyperpolarizing (Hyperpol.) E_{GABAAR} condition. Spiking activity was evoked by delivering brief depolarizing currents (input) of varying amplitudes to each neuron in the network.

(F) Raster plots for the same population of pyramidal neurons (n = 50) in the shunting (left) and hyperpolarizing (right) E_{GABAAR} conditions.

(G) Synchrony (top) and entropy (bottom) for pyramidal neurons in the shunting and hyperpolarizing E_{GABAAR} conditions (n = 1,000 randomly selected). Dashed line indicates the line of equality.

(H) Neurons in the shunting E_{GABAAR} condition exhibited lower synchrony (Shunt: 0.784 ± 0.001 vs. Hyperpol: 0.881 ± 0.001, $p < 0.001$, paired t test) and higher entropy (Shunt: 0.451 ± 0.002 nats vs. Hyperpol: 0.197 ± 0.002 nats, $p < 0.001$, paired t test) (n = 16,000 pyramidal neurons from the model). ***$p < 0.001$. MO, motor cortex; Stim., electrical stimulation; TH, thalamus.

responses were more closely aligned to the spiking of other neurons (Figure 4B). To quantify this, we (1) determined the degree of neuronal synchrony, which relates the firing of a single neuron to the activity of the other neurons on a trial-by-trial basis,[31,32] and (2) computed the peri-stimulus histogram entropy, as a measure of the flexibility (or variability) of each neuron's responses.[33] This revealed that in the awake state, stimulus-evoked synchrony was lower and entropy was higher (Figures 4C and 4D). As the mean firing rate was greater in the awake state, we also established that the differences in synchrony and entropy were still evident after regressing out the effect of firing rate (Figure S6).

To compliment these *in vivo* recordings, we constructed simple computational network models of recurrently connected excitatory pyramidal neurons and inhibitory interneurons. This enabled us to selectively vary synaptic E_{GABAAR} and thereby compare the effects of shunting and hyperpolarizing $GABA_AR$-mediated synaptic inhibition upon responses to excitatory input (Figure 4E). We observed that networks utilizing shunting E_{GABAAR} exhibited more variable input-evoked spike times, whereas networks with hyperpolarizing E_{GABAAR} exhibited less activity and the input-evoked spiking was more closely aligned to the spiking of other neurons in the network

CellPress
OPEN ACCESS

(Figure 4F). This was captured by the same measures that were applied to the *in vivo* recordings, revealing that shunting E_{GABAAR} was associated with lower synchrony and higher entropy, similar to what was observed in the awake state (Figures 4G and 4H). The higher entropy associated with shunting E_{GABAAR} would also be predicted to improve a network's ability to encode different stimuli. Indeed, when we assessed a network's ability to encode different patterned inputs, we observed that networks with shunting E_{GABAAR} could be more accurately decoded by a downstream classifier (Figure S7). Taken together, these observations support the idea that shunting inhibition promotes the desynchronization of local cortical networks and enables greater flexibility in terms of neuronal responses.

DISCUSSION

To determine how fast synaptic inhibition operates *in vivo*, we require measurements that preserve the ionic driving forces acting upon $GABA_A$Rs, while providing information that can relate a neuron's synaptic $GABA_A$R conductances, synaptic E_{GABAAR}, and V_m. Whole-cell patch-clamp recordings have revealed prominent $GABA_A$R synaptic conductances in awake cortex, but they compromise the ionic gradients underlying synaptic $GABA_A$R transmission.[3] Imaging approaches have provided estimates of intracellular Cl^- levels *in vivo*,[34,35] but they do not offer a readout of E_{GABAAR} as they do not account for extracellular ion concentrations or the permeability of the $GABA_A$ receptor; furthermore, they typically provide information on Cl^- throughout the soma, not at GABAergic synapses. Efforts have also been made to infer synaptic $GABA_A$R function from whole-cell recordings[22] or extracellular recordings,[36] although these approaches do not provide direct measurements of E_{GABAAR} or DF_{GABAAR}.

By combining *in vivo* gramicidin perforated recordings with optogenetic activation of synaptic $GABA_A$Rs, we have measured synaptic DF_{GABAAR} in the intact brain. While *in vivo* gramicidin recordings have been performed in early developing systems,[37,38] this is the first demonstration in the intact mammalian brain. Our recordings demonstrate that the nature of $GABA_A$R signaling is linked to the network's activity. Synaptic $GABA_A$Rs in awake cortex exhibit relatively depolarized E_{GABAAR} and low DF_{GABAAR}, such that their inhibitory effects are more likely to result from local changes in R_m (i.e., shunting effects). This provides experimental support for theoretical predictions regarding how E_{GABAAR} reflects a dynamic equilibrium between Cl^- extrusion and intrusion processes, and how Cl^- influxes associated with high $GABA_A$R activity can increase the Cl^- load experienced by a neuron.[9,27,28] Our observations have implications for how fast synaptic inhibition might vary under other *in vivo* network conditions that are associated with changes in ongoing synaptic activity,[24,39] and how such short-term, activity-dependent changes may interact with longer-term fluctuations in Cl^- homeostasis.[40] In addition, while acknowledging the space-clamp limitations associated with *in vivo* patch-clamp recordings,[41,42] future work could explore $GABA_A$R synapses in different neuronal compartments.

A synaptic E_{GABAAR} that favors shunting has important implications for neural computation. This includes effects upon synaptic integration,[13] gain modulation,[14] and network synchronization.[43] In the case of synaptic inhibition that is purely shunting, the duration of inhibition is restricted to the time course of the $GABA_A$R conductance. By contrast, hyperpolarizing $GABA_A$R-mediated inhibition is more long-lasting, meaning it can strongly synchronize the recovery of neurons within the network.[44] In addition to these temporal differences, the spatial effects of shunting inhibition depend upon where the $GABA_A$R conductance occurs within the neuron and with respect to other synaptic inputs, which introduces further diversity for neural computations.[13,45,46] Our analysis of *in vivo* spiking activity and network modeling experiments suggest that the shunting synaptic E_{GABAAR} observed in active awake cortical networks can promote a desynchronization of local networks and increase their ability to encode external inputs. These findings support the general idea that dynamic changes in $GABA_A$R-mediated synaptic inhibition can be used to optimize cortical function.

STAR★METHODS

Detailed methods are provided in the online version of this paper and include the following:

- KEY RESOURCES TABLE
- RESOURCE AVAILABILITY
  - Lead contact
  - Materials availability
  - Data and code availability
- EXPERIMENTAL MODEL AND SUBJECT DETAILS
- METHOD DETAILS
  - Surgical procedures
  - Electrophysiological recordings
  - Biocytin labelling
  - Neuronal network simulations
  - Simulating measurements of synaptic E_{GABAAR}
- QUANTIFICATION AND STATISTICAL ANALYSIS
  - Membrane potential measurements from current-clamp recordings
  - Background synaptic activity
  - Polarity of GABAergic responses
  - $GABA_A$ receptor equilibrium potential (E_{GABAAR})
  - Pre-processing of Neuropixels dataset
  - Characterization of stimulus-evoked spiking responses
  - Statistical analyses

SUPPLEMENTAL INFORMATION

Supplemental information can be found online at https://doi.org/10.1016/j.neuron.2023.08.005.

ACKNOWLEDGMENTS

We would like to thank members of the Akerman lab for advice and comments. We thank Mateo Velez-Fort, Christopher Pugh, and Troy Margrie (Sainsbury Wellcome Centre) for their help in setting up the *in vivo* patch-clamp

recordings. We thank Leslie Claar at the Allen Institute for making data publicly available, providing assistance with interpreting the data, and commenting on the results of our analyses. We thank Lex Kravitz, Luigi Petrucco, and Ethan Tyler for sharing their mouse illustrations on the Sci-Draw opensource platform and Ed Mann for his comments on the manuscript. The research leading to these results has received funding from the European Research Council under grant agreement 617670, plus the BBSRC project BB/S007938/1, and a research grant from the Leverhulme Trust. In addition, this work was supported by a Shaun Johnson Memorial Scholarship sponsored by the Leverhulme Trust and Mandela Rhodes Foundation (to R.J.B.) and a Wellcome Trust International Intermediate Fellowship 222968/Z/21/Z (to J.V.R.).

AUTHOR CONTRIBUTIONS

Conceptualization, R.J.B., P.J.N.B., and C.J.A.; methodology, R.J.B., P.J.N.B., and J.V.R.; investigation, R.J.B., P.J.N.B., and J.V.R.; writing – original draft, R.J.B., P.J.N.B., and C.J.A.; writing – review & editing, R.J.B., P.J.N.B., J.V.R., A.S., and C.J.A.; funding acquisition, R.J.B., A.S., and C.J.A.; and supervision, A.S. and C.J.A.

DECLARATION OF INTERESTS

The authors declare no competing interests.

INCLUSION AND DIVERSITY

We support inclusive, diverse, and equitable conduct of research.

Received: March 1, 2023
Revised: July 3, 2023
Accepted: August 4, 2023
Published: September 3, 2023

REFERENCES

1. Okun, M., and Lampl, I. (2008). Instantaneous correlation of excitation and inhibition during ongoing and sensory-evoked activities. Nat. Neurosci. *11*, 535–537. https://doi.org/10.1038/nn.2105.
2. Wehr, M., and Zador, A.M. (2003). Balanced inhibition underlies tuning and sharpens spike timing in auditory cortex. Nature *426*, 442–446. https://doi.org/10.1038/nature02116.
3. Haider, B., Häusser, M., and Carandini, M. (2013). Inhibition dominates sensory responses in the awake cortex. Nature *493*, 97–100. https://doi.org/10.1038/nature11665.
4. Atallah, B.V., and Scanziani, M. (2009). Instantaneous modulation of gamma oscillation frequency by balancing excitation with inhibition. Neuron *62*, 566–577. https://doi.org/10.1016/j.neuron.2009.04.027.
5. Takeuchi, A., and Takeuchi, N. (1967). Anion permeability of the inhibitory post-synaptic membrane of the crayfish neuromuscular junction. J. Physiol. *191*, 575–590. https://doi.org/10.1113/jphysiol.1967.sp008269.
6. Bormann, J., Hamill, O.P., and Sakmann, B. (1987). Mechanism of anion permeation through channels gated by glycine and gamma-aminobutyric acid in mouse cultured spinal neurones. J. Physiol. *385*, 243–286. https://doi.org/10.1113/jphysiol.1987.sp016493.
7. Kaila, K. (1994). Ionic basis of GABAA receptor channel function in the nervous system. Prog. Neurobiol. *42*, 489–537. https://doi.org/10.1016/0301-0082(94)90049-3.
8. Blaesse, P., Airaksinen, M.S., Rivera, C., and Kaila, K. (2009). Cation-chloride cotransporters and neuronal function. Neuron *61*, 820–838. https://doi.org/10.1016/j.neuron.2009.03.003.
9. Doyon, N., Vinay, L., Prescott, S.A., and De Koninck, Y. (2016). Chloride regulation: A dynamic equilibrium crucial for synaptic inhibition. Neuron *89*, 1157–1172. https://doi.org/10.1016/j.neuron.2016.02.030.
10. Düsterwald, K.M., Currin, C.B., Burman, R.J., Akerman, C.J., Kay, A.R., and Raimondo, J.V. (2018). Biophysical models reveal the relative importance of transporter proteins and impermeant anions in chloride homeostasis. eLife *7*, e39575. https://doi.org/10.7554/eLife.39575.
11. Fatt, P., and Katz, B. (1953). The effect of inhibitory nerve impulses on a crustacean muscle fibre. J. Physiol. *121*, 374–389. https://doi.org/10.1113/jphysiol.1953.sp004952.
12. Staley, K.J., and Mody, I. (1992). Shunting of excitatory input to dentate gyrus granule cells by a depolarizing GABAA receptor-mediated postsynaptic conductance. J. Neurophysiol. *68*, 197–212. https://doi.org/10.1152/jn.1992.68.1.197.
13. Gulledge, A.T., and Stuart, G.J. (2003). Excitatory actions of GABA in the cortex. Neuron *37*, 299–309. https://doi.org/10.1016/S0896-6273(02)01146-7.
14. Mitchell, S.J., and Silver, R.A. (2003). Shunting inhibition modulates neuronal gain during synaptic excitation. Neuron *38*, 433–445. https://doi.org/10.1016/S0896-6273(03)00200-9.
15. Thompson, S.M., Deisz, R.A., and Prince, D.A. (1988). Relative contributions of passive equilibrium and active transport to the distribution of chloride in mammalian cortical neurons. J. Neurophysiol. *60*, 105–124. https://doi.org/10.1152/jn.1988.60.1.105.
16. Rivera, C., Voipio, J., Payne, J.A., Ruusuvuori, E., Lahtinen, H., Lamsa, K., Pirvola, U., Saarma, M., and Kaila, K. (1999). The potassium chloride co-transporter KCC2 renders GABA hyperpolarizing during neuronal maturation. Nature *397*, 5. https://doi.org/10.1038/16697.
17. Doyon, N., Prescott, S.A., Castonguay, A., Godin, A.G., Kröger, H., and De Koninck, Y.D. (2011). Efficacy of synaptic inhibition depends on multiple, dynamically interacting mechanisms implicated in chloride homeostasis. PLoS Comput. Biol. *7*, e1002149. https://doi.org/10.1371/journal.pcbi.1002149.
18. Yelhekar, T.D., Druzin, M., and Johansson, S. (2017). Contribution of resting conductance, GABAA-receptor mediated miniature synaptic currents and neurosteroid to chloride homeostasis in central neurons. eNeuro *4*, 9–17. https://doi.org/10.1523/ENEURO.0019-17.2017.
19. Ebihara, S., Shirato, K., Harata, N., and Akaike, N. (1995). Gramicidin-perforated patch recording: GABA response in mammalian neurones with intact intracellular chloride. J. Physiol. *484*, 77–86. https://doi.org/10.1113/jphysiol.1995.sp020649.
20. Kyrozis, A., and Reichling, D.B. (1995). Perforated-patch recording with gramicidin avoids artifactual changes in intracellular chloride concentration. J. Neurosci. Methods *57*, 27–35. https://doi.org/10.1016/0165-0270(94)00116-x.
21. Kätzel, D., Zemelman, B.V., Buetfering, C., Wölfel, M., and Miesenböck, G. (2011). The columnar and laminar organization of inhibitory connections to neocortical excitatory cells. Nat. Neurosci. *14*, 100–107. https://doi.org/10.1038/nn.2687.
22. Valeeva, G., Tressard, T., Mukhtarov, M., Baude, A., and Khazipov, R. (2016). An optogenetic approach for investigation of excitatory and inhibitory network GABA actions in mice expressing Channelrhodopsin-2 in GABAergic neurons. J. Neurosci. *36*, 5961–5973. https://doi.org/10.1523/JNEUROSCI.3482-15.2016.
23. Davies, C.H., Davies, S.N., and Collingridge, G.L. (1990). Paired-pulse depression of monosynaptic GABA-mediated inhibitory postsynaptic responses in rat hippocampus. J. Physiol. *424*, 513–531. https://doi.org/10.1113/jphysiol.1990.sp018080.
24. Poulet, J.F.A., and Petersen, C.C.H. (2008). Internal brain state regulates membrane potential synchrony in barrel cortex of behaving mice. Nature *454*, 881–885. https://doi.org/10.1038/nature07150.
25. Constantinople, C.M., and Bruno, R.M. (2011). Effects and mechanisms of wakefulness on local cortical networks. Neuron *69*, 1061–1068. https://doi.org/10.1016/j.neuron.2011.02.040.
26. Destexhe, A., Rudolph, M., and Paré, D. (2003). The high-conductance state of neocortical neurons in vivo. Nat. Rev. Neurosci. *4*, 739–751. https://doi.org/10.1038/nrn1198.

CellPress
OPEN ACCESS

27. Staley, K.J., Soldo, B.L., and Proctor, W.R. (1995). Ionic mechanisms of neuronal excitation by inhibitory GABAA receptors. Science *269*, 977–981. https://doi.org/10.1126/science.7638623.

28. Staley, K.J., and Proctor, W.R. (1999). Modulation of mammalian dendritic GABA-A receptor function by the kinetics of chloride and bicarbonate transport. J. Physiol. *519*, 693–712. https://doi.org/10.1111/j.1469-7793.1999.0693n.x.

29. Claar, L.D., Rembado, I., Kuyat, J.R., Russo, S., Marks, L.C., Olsen, S.R., and Koch, C. (2023). Cortico-thalamo-cortical interactions modulate electrically evoked EEG responses in mice. Elife *12*, RP84630. https://doi.org/10.7554/eLife.84630.

30. Claar, L.D., Rembado, I., Kuyat, J.R., Russo, S., Marks, L.C., Olsen, S.R., and Koch, C. (2023). Simultaneous electroencephalography, extracellular electrophysiology, and cortical electrical stimulation in head-fixed mice. Version 0.230317.0039 [Dataset]. DANDI archive. https://doi.org/10.48324/dandi.000458/0.230317.0039.

31. Tsodyks, M., Kenet, T., Grinvald, A., and Arieli, A. (1999). Linking spontaneous activity of single cortical neurons and the underlying functional architecture. Science *286*, 1943–1946. https://doi.org/10.1126/science.286.5446.1943.

32. Bharioke, A., Munz, M., Brignall, A., Kosche, G., Eizinger, M.F., Ledergerber, N., Hillier, D., Gross-Scherf, B., Conzelmann, K.K., Macé, E., et al. (2022). General anesthesia globally synchronizes activity selectively in layer 5 cortical pyramidal neurons. Neuron *110*, 2024–2040.e10. https://doi.org/10.1016/j.neuron.2022.03.032.

33. Shannon, C.E. (1948). A mathematical theory of communication. Bell Syst. Tech. J. *27*, 379–423. https://doi.org/10.1002/j.1538-7305.1948.tb01338.x.

34. Sulis Sato, S., Artoni, P., Landi, S., Cozzolino, O., Parra, R., Pracucci, E., Trovato, F., Szczurkowska, J., Luin, S., Arosio, D., et al. (2017). Simultaneous two-photon imaging of intracellular chloride concentration and pH in mouse pyramidal neurons in vivo. Proc. Natl. Acad. Sci. USA *114*. E8770–E8779. https://doi.org/10.1073/pnas.1702861114.

35. Boffi, J.C., Knabbe, J., Kaiser, M., and Kuner, T. (2018). KCC2-dependent steady-state intracellular chloride concentration and pH in cortical Layer 2/3 neurons of anesthetized and awake mice. Front. Cell. Neurosci. *12*, 7. https://doi.org/10.3389/fncel.2018.00007.

36. Dubanet, O., Ferreira Gomes Da Silva, A., Frick, A., Hirase, H., Beyeler, A., and Leinekugel, X. (2021). Probing the polarity of spontaneous perisomatic GABAergic synaptic transmission in the mouse CA3 circuit in vivo. Cell Rep. *36*, 109381. https://doi.org/10.1016/j.celrep.2021.109381.

37. Zhang, R.W., Zhang, S.Y., and Du, J.L. (2013). KCC2-dependent subcellular ECl difference of ON-OFF retinal ganglion cells in larval zebrafish. Front. Neural Circuits *7*, 103. https://doi.org/10.3389/fncir.2013.00103.

38. van Rheede, J.J., Richards, B.A., and Akerman, C.J. (2015). Sensory-evoked spiking behavior emerges via an experience-dependent plasticity mechanism. Neuron *87*, 1050–1062. https://doi.org/10.1016/j.neuron.2015.08.021.

39. Steriade, M., McCormick, D.A., and Sejnowski, T.J. (1993). Thalamocortical oscillations in the sleeping and aroused brain. Science *262*, 679–685. https://doi.org/10.1126/science.8235588.

40. Alfonsa, H., Burman, R.J., Brodersen, P.J.N., Newey, S.E., Mahfooz, K., Yamagata, T., Panayi, M.C., Bannerman, D.M., Vyazovskiy, V.V., and Akerman, C.J. (2023). Intracellular chloride regulation mediates local sleep pressure in the cortex. Nat. Neurosci. *26*, 64–78. https://doi.org/10.1038/s41593-022-01214-2.

41. Williams, S.R., and Mitchell, S.J. (2008). Direct measurement of somatic voltage clamp errors in central neurons. Nat. Neurosci. *11*, 790–798. https://doi.org/10.1038/nn.2137.

42. To, M.S., Honnuraiah, S., and Stuart, G.J. (2022). Voltage clamp errors during estimation of concurrent excitatory and inhibitory synaptic input to neurons with dendrites. Neuroscience *489*, 98–110. https://doi.org/10.1016/j.neuroscience.2021.08.024.

43. Vida, I., Bartos, M., and Jonas, P. (2006). Shunting inhibition improves robustness of gamma oscillations in hippocampal interneuron networks by homogenizing firing rates. Neuron *49*, 107–117. https://doi.org/10.1016/j.neuron.2005.11.036.

44. Pavlov, I., Scimemi, A., Savtchenko, L., Kullmann, D.M., and Walker, M.C. (2011). Ih-mediated depolarization enhances the temporal precision of neuronal integration. Nat. Commun. *2*, 199. https://doi.org/10.1038/ncomms1202.

45. Hao, J., Wang, X.D., Dan, Y., Poo, M.M., and Zhang, X.H. (2009). An arithmetic rule for spatial summation of excitatory and inhibitory inputs in pyramidal neurons. Proc. Natl. Acad. Sci. USA *106*, 21906–21911. https://doi.org/10.1073/pnas.0912022106.

46. Paulus, W., and Rothwell, J.C. (2016). Membrane resistance and shunting inhibition: where biophysics meets state-dependent human neurophysiology. J. Physiol. *594*, 2719–2728. https://doi.org/10.1113/JP271452.

47. Harden, S.W. (2022). pyABF 2.3.5. [Online]. Available: https://pypi.org/project/pyabf

48. Margrie, T.W., Brecht, M., and Sakmann, B. (2002). In vivo, low-resistance, whole-cell recordings from neurons in the anaesthetized and awake mammalian brain. Pflugers Arch. *444*, 491–498. https://doi.org/10.1007/s00424-002-0831-z.

49. Wang, Y., Liu, Y.Z., Wang, S.Y., and Wang, Z. (2016). In vivo whole-cell recording with high success rate in anaesthetized and awake mammalian brains. Mol. Brain *9*, 86. https://doi.org/10.1186/s13041-016-0266-7.

50. Jouhanneau, J.S., and Poulet, J.F.A. (2019). Multiple two-photon targeted whole-cell patch-clamp recordings from monosynaptically connected neurons in vivo. Front. Synaptic Neurosci. *11*, 15. https://doi.org/10.3389/fnsyn.2019.00015.

51. Jordan, R. (2021). Optimized protocol for in vivo whole-cell recordings in head-fixed, awake behaving mice. Star Protoc. *2*, 100347. https://doi.org/10.1016/j.xpro.2021.100347.

52. Delpire, E., Baranczak, A., Waterson, A.G., Kim, K., Kett, N., Morrison, R.D., Daniels, J.S., Weaver, C.D., and Lindsley, C.W. (2012). Further optimization of the K-Cl cotransporter KCC2 antagonist ML077: development of a highly selective and more potent in vitro probe. Bioorg. Med. Chem. Lett. *22*, 4532–4535. https://doi.org/10.1016/j.bmcl.2012.05.126.

53. Sheardown, M.J., Nielsen, E.O., Hansen, A.J., Jacobsen, P., and Honoré, T. (1990). 2,3-Dihydroxy-6-nitro-7-sulfamoyl-benzo(F)quinoxaline: a neuroprotectant for cerebral ischemia. Science *247*, 571–574. https://doi.org/10.1126/science.2154034.

54. Lundbaek, J.A., Maer, A.M., and Andersen, O.S. (1997). Lipid bilayer electrostatic energy, curvature stress, and assembly of gramicidin channels. Biochemistry *36*, 5695–5701. https://doi.org/10.1021/bi9619841.

55. Traynelis, S.F. (1998). Software-based correction of single compartment series resistance errors. J. Neurosci. Methods *86*, 25–34. https://doi.org/10.1016/S0165-0270(98)00140-X.

56. Stimberg, M., Brette, R., and Goodman, D.F. (2019). Brian 2, an intuitive and efficient neural simulator. eLife *8*, e47314. https://doi.org/10.7554/eLife.47314.

57. Vogels, T.P., Sprekeler, H., Zenke, F., Clopath, C., and Gerstner, W. (2011). Inhibitory plasticity balances excitation and inhibition in sensory pathways and memory networks. Science *334*, 1569–1573. https://doi.org/10.1126/science.1211095.

58. Hines, M.L., and Carnevale, N.T. (1997). The NEURON simulation environment. Neural Comput. *9*, 1179–1209. https://doi.org/10.1162/neco.1997.9.6.1179.

59. Ascoli, G.A., Donohue, D.E., and Halavi, M. (2007). NeuroMorpho.Org: a central resource for neuronal morphologies. J. Neurosci. *27*, 9247–9251. https://doi.org/10.1523/JNEUROSCI.2055-07.2007.

60. Madisen, L., Garner, A.R., Shimaoka, D., Chuong, A.S., Klapoetke, N.C., Li, L., van der Bourg, A., Niino, Y., Egolf, L., Monetti, C., et al. (2015). Transgenic mice for intersectional targeting of neural sensors and effectors with high specificity and performance. Neuron *85*, 942–958. https://doi.org/10.1016/j.neuron.2015.02.022.

61. Virtanen, P., Gommers, R., Oliphant, T.E., Haberland, M., Reddy, T., Cournapeau, D., Burovski, E., Peterson, P., Weckesser, W., Bright, J., et al. (2020). SciPy 1.0: fundamental algorithms for scientific computing in Python. Nat. Methods *17*, 261–272. https://doi.org/10.1038/s41592-019-0686-2.

CellPress
OPEN ACCESS

STAR★METHODS

KEY RESOURCES TABLE

REAGENT or RESOURCE	SOURCE	IDENTIFIER
**Antibodies**		
Streptavidin-Cy3	ThermoFisher	438315
**Chemicals, peptides, and recombinant proteins**		
Biocytin	Merck	576-19-2
NBQX (2,3-dihydroxy-6-nitro-7-sulfamoylbenzo (F) quinoxaline)	Torcis	1044
Urethane	Merck	U2500
VU0463271	Torcis	4719
**Deposited data**		
Neuropixels dataset	Claar et al. [29,30]	DANDI Archive: 000458
**Experimental models: Organisms/strains**		
Gad2-IRES-Cre mice	Jackson Labs	RRID:IMSR_JAX:010802
Ai32(RCL-ChR2(H134R)/EYFP)	Jackson Labs	RRID:IMSR_JAX:012569
**Software and algorithms**		
Brian Simulator	https://briansimulator.org/	RRID:SCR_002998
ImageJ	https://imagej.net/	RRID:SCR_003070
MATLAB	MathWorks	RRID:SCR_001622
NEURON	http://www.neuron.yale.edu	RRID:SCR_005393
NumPy	http://www.numpy.org	RRID:SCR_008633
pCLAMP 11 Software	Molecular Devices	RRID:SCR_011323
pyABF 2.3.7	Harden (2022)[47]	https://github.com/swharden/pyABF
Python 3.10.9	https://www.python.org/	RRID:SCR_008394
Scipy	http://www.scipy.org/	RRID:SCR_008058
ZEISS Imaging Suite	http://www.zeiss.com	RRID:SCR_013672
**Other**		
Custom code (Python)	This paper	https://gist.github.com/paulbrodersen
Custom code (Matlab)	This paper	https://github.com/richardjburman
Digidata 1550B Data Acquisition System	Molecular Devices	Digidata 1550B1
HumBug noise eliminator (50Hz)	Digitimer	SKU: D.HUMBUG
LSM 880 microscope	Zeiss	-
MultiClamp 700B Microelectrode Amplifier	Molecular Devices	N/A
Optopatcher	A-M Systems	663849

RESOURCE AVAILABILITY

Lead contact

Further information and requests for resources and reagents should be directed to and will be fulfilled by the lead contact, Colin Akerman (colin.akerman@pharm.ox.ac.uk).

Materials availability

No new materials were generated in this study.

Data and code availability

Custom code used in analysis of data is publicly available as of the date of publication (links provided in the key resources table). Any additional materials required to reanalyze data are available on request to the lead contact.

EXPERIMENTAL MODEL AND SUBJECT DETAILS

All mice were bred, housed and used in accordance with the United Kingdom Animals (Scientific Procedures) Act (1986). Homozygous Gad2-IRES-Cre mice were crossed with homozygous Ai32(RCL-ChR2(H134R)/EYFP) mice. This produced a heterozygous colony expressing channelrhodopsin-2 (ChR2(H134R)-YFP) in Gad2-positive neurons, which includes the main subclasses of GABAergic interneurons.[21] Mice were purchased from Jackson Laboratory (Maine, USA). Both male and female mice were used in the experiments. Mice were maintained under a 12-hour:12-hour light-dark cycle and fed ad libitum. For all experiments both males and females were used, and mice were between six to eight weeks postnatal age at the time of recording.

METHOD DETAILS

Surgical procedures

The preparation for anesthetized recordings was adapted from previously published protocols.[48–50] Mice were anesthetized with an intraperitoneal (IP) injection of 25 % urethane (1 g/kg, diluted in sterile PBS). To counteract adverse events caused by urethane, a bolus of the anticholinergic agent, Glycopyrronium Bromide (0.01 mg/kg) was administered subcutaneously (SC). Local anesthetic (Marcain 2 mg/kg) was applied intradermally to the scalp and topically in the ears prior to mounting the mouse into the head holding apparatus (Narishige) under a surgical stereoscope (Olympus). The mouse's body temperature was maintained at 37 degrees Celsius using a heating mat and rectal probe. The animal's head was shaved and eye-protecting ointment (Viscotears) was applied to both eyes. An incision in the scalp was made using surgical scissors and the area expanded with blunt dissection to expose the skull. The site of the craniotomy was marked over the primary visual cortex (V1). Tissue adhesive (Vetbond) was applied to fix the surrounding scalp to the skull and to secure cranial sutures. Multiple layers of dental cement (Simplex Rapid) were applied to create a recording chamber on top of the skull. A 0.5 mm craniotomy was drilled over the marked region using a dental drill (Foredom). The craniotomy was submerged in cortex buffer (containing, in mM: 125 NaCl, 5 KCl, 10 HEPES, 2 $MgSO_4 \cdot 7H_2O$, 2 $CaCl_2 \cdot 2H_2O$, 10 Glucose). The bone flap and dura were removed. The animal was then transferred to the *in vivo* patch setup and the recording session typically lasted 3 hours between zeitgeber time 3 (ZT3) and ZT6, at which point the animal was culled. Throughout the recording session, the depth of anesthesia was monitored by testing for pedal reflexes.

The preparation for awake recordings consisted of three phases based on published protocols.[48,51] The first phase involved the fixation of the head plate. Mice were anesthetized with isoflurane (Zoetis) and mounted into a stereotaxic frame (Kopf). Subcutaneous analgesia (meloxicam 5mg/kg and buprenorphine 0.1mg/kg) was administered along with intradermal local analgesic (marcain 2 mg/kg) into the scalp. The scalp was shaved (Wahl) and cleaned (Hibiscrub). Eye-protecting ointment (Viscotears) was applied. The scalp was then removed and the site was washed with sterile cortex buffer (containing, in mM: 125 NaCl, 5 KCl, 10 HEPES, 2 $MgSO_4 \cdot 7H_2O$, 2 $CaCl_2 \cdot 2H_2O$, 10 Glucose). After drying the skull with adsorbent swabs (Haag-Streit), the periosteum was removed using a micro curette (Fine Science Tools). Tissue adhesive (Vetbond) was applied to secure cranial sutures and to fix the surrounding scalp to the underlying bone. A custom-designed aluminum headplate with a 7 mm well was bonded to the skull, first with adhesive glue (Loctite), and then followed by serial layers of dental cement (Super-Bond). The well was then covered with silicone sealant (Kwik-Cast). The animal was singly housed and allowed to recover. From day three following head plate fixation, the animal was habituated to head-fixation for increasing time intervals up to 60 minutes. On the day of the recording, the animal was briefly anesthetized with isoflurane and mounted onto a stereotaxic frame. The silicone sealant was removed and the area washed with sterile cortex buffer. A 0.5 mm craniotomy was created using a dental drill (Foredom) and the bone flap removed. A durectomy was performed and the site was covered with a soft dressing soaked in cortex buffer. This step in the procedure was limited to 20 minutes. The animal was then remounted onto the head-fixation setup and transferred to the *in vivo* patch setup. The mouse was allowed to fully recover for at least 30 minutes before recording was commenced. Recording sessions typically lasted 2-3 hours between ZT3 and ZT6, at which point the animal was culled. During the recording sessions, the experimenter visually monitored the mouse to confirm that the animal was awake.

Electrophysiological recordings

All electrophysiological recordings were performed using a Multiclamp 700B amplifier (Molecular Devices) and digitized at 20 kHz (Digidata 1550, Molecular Devices). A HumBug noise eliminator (Digitimer) was used to remove 50 Hz noise. To perform whole-cell patch clamp recordings, patch pipettes were back-filled with K^+ methanesulfonate internal solution (in mM: 110 KMeSO3, 6 NaOH, 3 $MgCl_2 \cdot 6H_2O$, 0.02 $CaCl_2$, 40 HEPES, 0.05 EGTA, 2 Na_2ATP, 0.5 NaGTP, 2 MgATP, 10 Biocytin). To perform perforated patch clamp recordings, the internal pipette solution was prepared immediately prior to recording by combining a high chloride (150 mM) solution (in mM: 141 KCl, 9 NaCl, 10 HEPES) heated to 37 degrees Celsius, with a stock solution of gramicidin A (4 mg/ml - dissolved in dimethyl sulfoxide, DMSO, Merck) to achieve a final concentration of 80 μg/mL gramicidin.[20] The solution was then vortexed (40 seconds) and sonicated (20 seconds). The patch pipette was back-filled with the gramicidin solution and mounted on a Optopatcher pipette holder (A-M Systems) which contained a 50 μm fiber (Thorlabs) connected to a 473 nm laser (MBL-FN-473-150mW, CNI Laser). Pipettes were lowered onto the brain surface and blind patching commenced. Once the gigaseal had formed, perforation was then monitored by observing changes in series resistance. Recording protocols were started once the series resistance had stabilized at <100 MΩ. In our experience, recordings that failed to reach these series resistance values within

the first 10 minutes never achieved a perforation quality that was suitable for recordings. Rupture or breakthrough of the perforation in to whole-cell configuration was detected by a sudden and persistent depolarization of the equilibrium potential of the $GABA_AR$ (E_{GABAAR}), consistent with dialysis of the neuron with the high chloride pipette solution. In a subset of experiments, KCC2 was blocked by injecting the selective antagonist, VU0463271,[52] directly into the cortex. The injection pipette contained 100 μM VU0463271 (Tocris) in ACSF, which was delivered at a rate of 33 nL/min to a total volume of 200 nL. In a subset of experiments where local network activity was reduced, the α-amino-3-hydroxy-5-methyl-4-isoxazolepropionic acid (AMPA) receptor blocker 2,3-dihydroxy-6-nitro-7-sulfamoylbenzo (F) quinoxaline (NBQX) was injected directly into the cortex.[53] The injection pipette contained 100 μM NBQX (Tocris) in ACSF, which was delivered at a rate of 33 nL/min to a total volume of 250 nL.

Data was acquired using recording protocols configured in Clampex (Molecular Devices) and analyzed using custom code written in Python. Online series resistance compensation was not used, as the high amounts of activity in the *in vivo* brain would cause large fluctuations in input current, which increases the rate of perforation rupture.[28,54] Therefore, to correct for series resistance effects, offline correction was performed.[55] The voltage drop caused by the series resistance (R_s) was calculated by multiplying the measured current response with 90 % of the R_s. The voltage drop was then subtracted from the command voltage to estimate the neuron's membrane potential. Membrane and recording properties were calculated by measuring the change in current in response to a -10 mV step during voltage clamp recordings. R_s was calculated from both the peak current elicited by the -10 mV voltage step, and by estimating the peak after fitting an exponential to the decay of the current transient response to the -10 mV step. These methods gave similar values and so the numerical average was used as a final estimate of R_s. To calculate the membrane resistance (R_m), R_s was subtracted from the measured input resistance.

Biocytin labelling

For reconstruction of biocytin-filled neurons, mouse brains were fixed via transcardial perfusion of phosphate buffered solution (PBS, 0.1 M) and 4 % paraformaldehyde solution (PFA). Brains were stored for 24 hours at 4°C in 4 % PFA and then washed and stored in PBS containing 0.05 % sodium-azide. Within a week of perfusion, brains were washed in PBS and mounted onto a microtome (HM650V, ThermoScientific) before being sectioned into 100 μm thick coronal slices whilst bathed in PBS. For biocytin labelling, sections were incubated in Streptavidin-Cy3 (1:1000, Thermo Fisher) for 2 hours at room temperature before being mounted with Vectashield (Vectorlabs) onto glass slides (Avantor). Images were acquired using a LSM 880 microscope (Zeiss). All images were captured using a 20x water-immersion objective (W Plan-Apochromat NA 1.0) through the ZEN software (Zeiss). Image processing was performed in ImageJ software (NIH).

Neuronal network simulations

Computational models were used to explore the effect of synaptic E_{GABAAR} upon neuronal synchrony, entropy, and stimulus discriminability. Neuronal networks were constructed using the neuron simulator Brian 2.[56] Each comprised 800 glutamatergic neurons and 200 GABAergic neurons. Each neuron was modelled as a single compartment, current-based leaky integrate-and-fire neuron. Free parameters were set as shown in Table 1. Neurons were connected to each other with a probability set uniformly to 10 %. Glutamatergic synaptic weights were set to 0.1 nS and GABAergic synaptic weights were initialized at 1 nS. Before running a simulation, homeostatic inhibitory synaptic plasticity was used to establish a balance of excitatory and inhibitory inputs to each neuron,[57] and then synaptic weights were frozen.

Table 1. Free parameters used for simple neuronal network modeling

Parameter	Set value
Equilibrium potential of the leak current (E_{Leak}, equivalent to RMP)	−60 mV
Equilibrium potential of the glutamatergic current	0 mV
Excitatory postsynaptic conductance decay time constant	5 ms
Inhibitory postsynaptic conductance time constant	10 ms
Membrane capacitance	200 pF
Membrane time constant	5 ms
Refractory period	5 ms

Synaptic E_{GABAAR} in glutamatergic neurons was set to -60 mV for the first half of each simulation and to -80 mV for the remainder, to represent the synaptic E_{GABAAR} recorded experimentally in the awake and anesthetized states, respectively. E_{GABAAR} was set to -60 mV in the GABAergic neurons. All external inputs were modelled as random, independent currents with values drawn from log-normal distributions with the scale parameter mu set to zero, and the shape parameter sigma set to 1.

$$X = M * \exp(\mu + \sigma * Z)$$

Z represents a standard normal variable. For the stimulus inputs, we generated 100 distinct input patterns. Each pattern consisted of a set of 1 ms long depolarizing current pulses delivered simultaneously, but with variable amplitudes (M = 500 pA), to each neuron in the network. The delivery of each input pattern was followed by a 25 ms long pause and each pattern was presented 100 times in each condition (shunting or hyperpolarizing E_{GABAAR}). To prevent full synchronization of all neurons, each neuron independently also received a noise input with variable amplitude (M = 25 pA), varied every 10 ms. Each simulation was repeated 20 times.

Simulating measurements of synaptic E_{GABAAR}

To investigate the effect of intrinsic membrane properties on our estimates of synaptic E_{GABAAR} under different network conditions, a multi-compartment model of an adult pyramidal neuron from L2/3 of mouse primary visual cortex was constructed using the NEURON simulation environment.[58] The neuron's morphology was sourced from NeuroMorpho[59] and based on a reconstruction (NMO_62358) that was shared by Madisen and colleagues.[60] Model parameters are shown in Table 2.

Table 2. Parameters used for modeling effects of membrane resistance

Parameter	Set value
Membrane capacitance	C_m = 2.515 μF/cm^2
Axial resistance (R_{axial})	150 Ωcm
Passive membrane reversal (equivalent to RMP)	An. e_{pas} = −70.7 mV<br>Aw. e_{pas} = −64.2 mV<br>+NBQX e_{pas} = −71.4 mV
Voltage clamp electrode series resistance (R_s)	47.5 MΩ
Passive membrane resistance	An. R_m = 10^3.784 Ωcm^2<br>Aw. R_m = 10^3.540 Ωcm^2<br>+NBQX R_m = 10^3.988 Ωcm^2

With these parameters, the membrane resistance (R_m) measured by simulated voltage clamp at the soma was 87.2 MΩ in the anesthetized condition, 50.6 MΩ in the awake condition, and 136.2 MΩ in the Awake + NBQX condition. These values matched the average experimentally measured membrane resistance from data (Figure S3). Activation of $GABA_ARs$ was simulated by placing twenty $GABA_AR$-containing synapses randomly within a 75 μm radius of the center of the soma. E_{GABAAR} was set to between -85 mV and -35 mV (iterated by 5 mV for each simulation). Activation of $GABA_AR$ synapses was simulated by using an alpha function with a tau of 150 ms. The peak local conductance of each $GABA_AR$ synapse was set to 2 nS. To simulate the experimental estimation of synaptic E_{GABAAR}, a simulated voltage clamp was placed at the soma. Two consecutive voltage ramps were then applied, one before and one during simulated activation of $GABA_ARs$ (to reproduce the experimental protocol). Synaptic E_{GABAAR} was estimated using IV plots, either with 0% R_s correction, or 90 % R_s correction, to replicate the experimental data acquisition and analysis process.

QUANTIFICATION AND STATISTICAL ANALYSIS

Membrane potential measurements from current-clamp recordings

To determine the resting membrane potential (RMP) during current clamp recordings, spontaneous activity was recorded for a continuous period of five minutes. During analysis, membrane potential values greater than -40 mV were removed to avoid distortions caused by action potentials. Average distribution plots were created by concatenating all membrane potential values across all neurons in each group, to which a Gaussian kernel-density estimate was then fitted.

Background synaptic activity

The level of subthreshold synaptic activity was determined by measuring the rate of change in the membrane potential over time, after excluding action potentials. The rate of change in membrane potential was calculated by taking the first derivative (V_m dV/dt in mV/ms). The first derivative was winsorized and the mean of the modulus of the V_m dV/dt calculated for each neuron.

Polarity of GABAergic responses

Synaptic $GABA_AR$-mediated responses in current clamp were evoked with a 10 ms light pulse, presented during a one second sweep. The average of 15 sweeps was then calculated and normalized to the mean membrane potential during the 100 ms preceding the light pulse. Current clamp recordings from a subset of the neurons in the NBQX condition also contributed to another study.[40] The polarity of the synaptic $GABA_AR$ response was defined as the mean normalized membrane potential during the 100 ms following the light pulse. If the mean value was greater than zero, the response was classified as depolarizing, whereas a mean value below zero was classified as hyperpolarizing.

GABA$_A$ receptor equilibrium potential (E_{GABAAR})

Two types of voltage clamp protocol were used to measure synaptic E_{GABAAR}: a ramp protocol and a step protocol. The ramp protocol involved clamping the neuron at -70 mV, and then imposing two consecutive voltage ramps, each lasting 150 ms, which extended from 60 mV below the holding voltage to 40 mV above the holding voltage (i.e. from -130 mV to -30 mV, at a rate of 0.7 mV/ms). The first ramp (i.e. the 'baseline' ramp) sampled the neuron's intrinsic membrane currents and the second ramp (i.e. the 'light' ramp) included a light-evoked synaptic GABA$_A$R conductance. The light-evoked synaptic GABA$_A$R conductance was elicited with a 10 ms light pulse that coincided with the start of the ramp, to ensure the evoked GABA$_A$R current was at its peak during the ramp.[23] During analysis, the first and last 15 ms of each ramp were excluded to avoid transient currents caused by the discharge of the pipette capacitance. The currents from both ramps were then superimposed on a current-voltage (IV) plot using the series corrected membrane potential and, to avoid action potentials and capacitance transients, the current responses were cropped to only include regions that were clear of these sources of contamination. A straight line was fitted to both currents. The current from the baseline ramp was used to infer the equilibrium potential of the holding current (from which the resting membrane potential, RMP, could be inferred), defined as the voltage at which the fitted line was equal to zero. The point at which the fitted lines for the two ramps intersected is E_{GABAAR}. This can also be calculated by subtracting the current response during the baseline ramp, from the current response during the light ramp. The voltage at which a fitted line to this subtracted current is equal to zero, is equivalent to E_{GABAAR}. The driving force can then be calculated by subtracting the measured E_{GABAAR} from the RMP. The slope of the subtracted current is equal to the GABA$_A$R conductance.

The step protocol meanwhile, involved estimating synaptic E_{GABAAR} from a voltage clamp protocol in which the neuron was exposed to a series of voltage steps, whilst eliciting a light-evoked GABA response during each step. The voltage was stepped in 10 mV increments from -130 mV to -30 mV. Each step lasted 500 ms, with a 10 s interval between steps. The light-evoked synaptic GABA conductance was evoked 100 ms after the start of each voltage step, by delivering a 100 ms light pulse. For analysis purposes, the membrane current was measured immediately before the light pulse (i.e. 'baseline' current) and then 20 ms after the onset of the light pulse, which corresponds to the peak of the GABA$_A$R conductance[23] (i.e. 'light' current, $Step_{20ms}$). A further current measurement was made at the time when E_{GABAAR} was estimated from the ramp protocol in the same cell ($Step_{Ramp}$). The currents were then used to estimate E_{GABAAR}, in a similar way to that described for the ramp protocol. The difference between the two E_{GABAAR} values (E_{GABAAR} at $Step_{20ms}$ and E_{GABAAR} at $Step_{Ramp}$) allowed us to assess the stability of E_{GABAAR} and the contribution of GABA$_B$Rs to our estimates of E_{GABAAR}, as previous described.[23]

Pre-processing of Neuropixels dataset

We analyzed a recent dataset, made publicly available by the Allen Institute.[30] Surgical procedures, habituation, Neuropixels recordings, electrical stimulation, and data processing including spike sorting are described in the manuscript accompanying the dataset.[29] Briefly, up to three Neuropixels probes were inserted per animal in order to target cortical regions and thalamic nuclei of interest. Electrical stimuli were delivered via a bipolar platinum-iridium stereotrode, which was located within 0.5 mm of the Neuropixels probe and targeted either layer 5/6 or layer 2/3 of the respective area of cortex. From the resulting dataset, we sub-selected all neurons that were in somatosensory cortex (annotations "SSp-ll" & "SSp-tr") or secondary motor cortex (annotations "MOs"), depending on which of the two areas had been electrically stimulated. As in Claar et al.,[29] regular spiking neurons were identified as having a waveform duration $\geq$ 0.4 ms. The evoked firing rate, synchrony, and peri-stimulus histogram entropy were computed using spikes that occurred within an interval of 2–12 ms following an electrical stimulus. The start and end of this interval were chosen to remove artifacts arising from the electrical stimulation and to exclude spikes elicited by recurrent activity from the thalamus, respectively.[29] Neurons with an average evoked firing rate below 0.1 Hz or above 200 Hz in either condition (awake/anesthesia) were excluded from further analysis (203 neurons). The 662 remaining neurons derived from 17 recordings in 16 mice, each contributing a median of 34 neurons (minimum: 1, maximum: 93).

Characterization of stimulus-evoked spiking responses

We applied measures of synchrony and entropy to describe the stimulus-evoked spiking activity from the Neuropixels *in vivo* recordings. To quantify the degree to which each neuron was in synchrony with the neuronal population as a whole, we adapted a measure used in Bharioke et al.[32] For each stimulus, we computed the population response by constructing the peri-stimulus spike histogram across all recorded neurons during the 12 ms following stimulus onset, using a bin width of 1 ms. For each neuron, we then computed the average magnitude of the population response each time the neuron spiked. Our measure differed from its previous definition in as much as we normalized each stimulus-evoked population response by the total spike count for the corresponding trial rather than the average firing rate. To quantify the variance in each neuron's stimulus-evoked responses, we computed the peri-stimulus spike histogram for the 12 ms following stimulus onset, using a bin width of 1 ms. We then calculated the normalized histogram's entropy using the standard formula for discrete variables,[33] as implemented in the scipy.stats python module.[61] The same measures of synchrony and entropy were used to describe the input-evoked spiking activity in the neuronal network simulations. To determine the separability of population responses to different external inputs in the neuronal network simulations, we stimulated the network using 100 different input patterns, each repeated 100 times. For each trial we computed the spike count in 50 randomly chosen neurons. Using the resulting 10000 population vectors we then trained and tested a k-nearest neighbor classifier using 5-fold stratified cross-validation.

Statistical analyses

All data is reported as mean ± standard error of the mean (SEM). For comparative statistics, the distribution of the continuous data was first established using the Shapiro-Wilk test for normality, which guided the subsequent use of appropriate parametric and non-parametric tests. For data used in both Figures 2 and 3, a one-way ANOVA with post-hoc correction with the Bonferroni method was used. All statistical analyses was performed using the Python SciPy library (provided in key resources table). Details of the data values, sample sizes and statistical measurements are provided in the figure legends.

Neuron, Volume *111*

Supplemental information

Active cortical networks promote shunting fast synaptic inhibition *in vivo*

Richard J. Burman, Paul J.N. Brodersen, Joseph V. Raimondo, Arjune Sen, and Colin J. Akerman

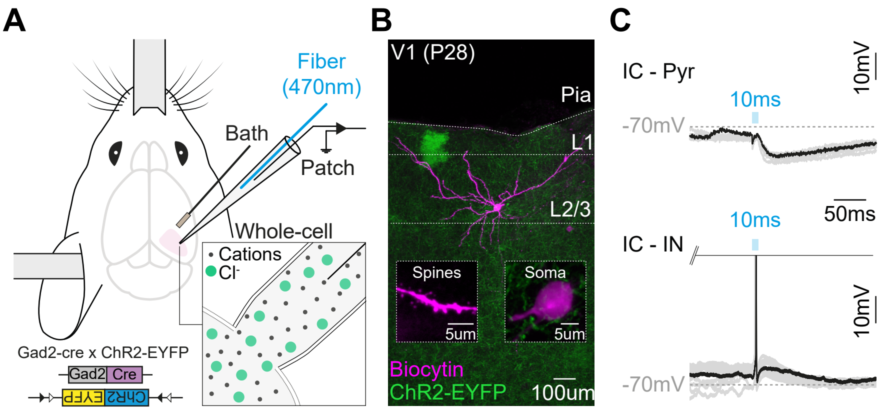

Supplementary Figure 1: Optical activation of Gad2-positive interneurons evokes post-synaptic GABA receptor responses in layer 2/3 cortical pyramidal neurons *in vivo*, related to Figure 1.

(**A**) Schematic of whole-cell patch clamp recording in primary visual cortex (V1) of a transgenic mouse expressing channelrhodopsin-2 fused to enhanced yellow fluorescent protein (ChR2-EYFP) in Gad2-positive interneurons. The Gad2-positive interneurons express Cre-recombinase and are therefore able to remove loxP sites controlling ChR2-YFP expression. Using this system it is possible to selectively express ChR2-YFP only in Gad2-positive GABA-releasing interneurons. To activate ChR2, blue laser light pulses were delivered via a 50 µm optical fibre that was positioned inside and towards the tip of the pipette. This allows for activation of ChR2-expressing pre-synaptic neurons that synapse onto the recorded neuron. Inset shows the whole-cell recording configuration, which allowed biocytin to enter the patched neuron from the internal pipette solution. (**B**) Confocal image of a biocytin-filled layer 2/3 pyramidal neuron stained with streptavidin-fluorophore conjugate, streptavidin-CY3. Inserts show magnified images of the spines and soma of the biocytin-filled neuron. (**C**) Current clamp recordings (‘IC’) of a light-evoked post-synaptic response (10 ms blue light illumination) from a pyramidal neuron (top, Pyr) and a ChR2-expressing Gad2-positive interneuron (bottom, IN). This meant that it was immediately apparent if we had patched a ChR2-expressing interneuron, as light pulses would elicit action potentials in the recorded neuron.

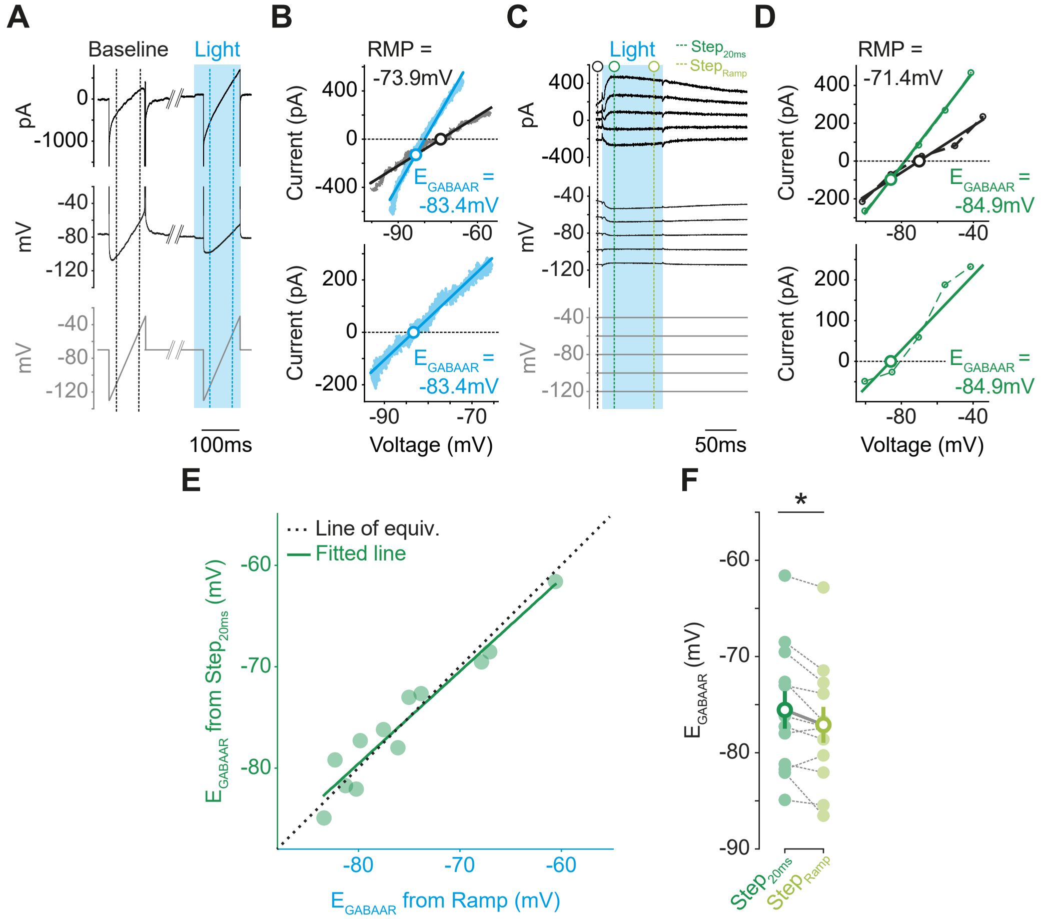

Supplementary Figure 2: *In vivo* voltage ramp protocols provide accurate estimates of synaptic E_{GABAAR}, related to Figure 1.

(**A**) Ramp protocol showing the command holding voltage (bottom, grey), the voltage after applying series resistance correction (R_s = 52.1 MΩ, middle, black), and the current response (black, top). Protocol consisted of a control ramp ('baseline') and a second ramp during which the light-evoked postsynaptic GABA$_A$R-mediated synaptic current was evoked with a blue light pulse ('light'; cyan shaded area). The pairs of vertical dashed lines indicate the regions of the ramps that were analyzed. (**B**) IV plots from a ramp protocol (top) in which the baseline (black) and light (cyan) current responses are shown. The intersection of the two fitted lines represents synaptic E_{GABAAR}, while the point at which the baseline current crosses zero is equivalent to the neuron's RMP. IV plot of the subtracted current (bottom), which corresponds to the synaptic GABA$_A$R current and has a value of zero at E_{GABAAR}. (**C**) A voltage step protocol was used to estimate synaptic E_{GABAAR} in the same neuron as in 'A'. The voltage step protocol (bottom, grey), the voltage after applying series resistance correction (R_s = 50.8 MΩ, middle, black) and current response (top, black) are shown. GABA$_A$R-mediated currents were evoked using light pulses (470 nm, 100 ms, cyan shading). Black vertical dashed line indicates where baseline current was calculated. Dark green vertical dashed line indicates measurement made at the peak of the pure GABA$_A$R-mediated synaptic current, 20 ms from light onset (Step$_{20ms}$).[23] Light green vertical dashed line indicates where measurement was made at the time E_{GABAAR} was estimated from the ramp protocol in the same cell (Step$_{Ramp}$). (**D**) IV plot (top) of the baseline current (black) and the current at Step$_{20ms}$ (dark green). The resulting subtracted current (bottom), corresponds to the synaptic GABA$_A$R current and has a value of zero at E_{GABAAR}. (**E**) A strong correlation was observed between synaptic E_{GABAAR} estimated from the ramp protocol (cyan) and from the voltage step protocol (dark green, Step$_{20ms}$) (*R squared* = 0.92, $p < 0.001$, n = 12 neurons from 6 mice, *Wald test*). (**F**) Population data showing a small (mean amplitude 1.5 mV), but statistically significant difference in E_{GABAAR} measurements between Step$_{20ms}$ and Step$_{Ramp}$ (Step$_{20ms}$: -75.55 ± 1.95 mV vs. Step$_{Ramp}$: -77.10 ± 1.86 mV, p = 0.01, *paired t-test*), consistent with good estimates of E_{GABAAR} during the ramp protocol and a modest potential contribution by GABA$_B$Rs.[1] *, $p < 0.05$.

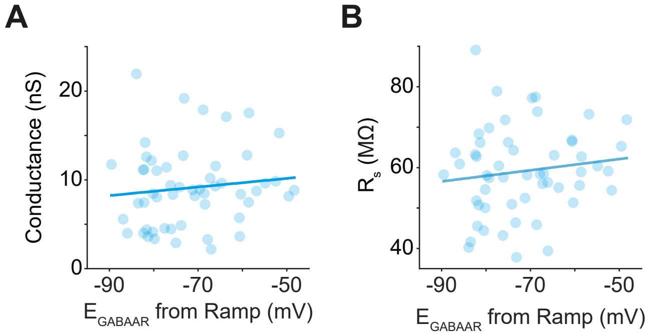

Supplementary Figure 3: Estimates of synaptic E_{GABAAR} are not related to the amplitude of the light-evoked postsynaptic GABA conductance or the series resistance, related to Figure 1.

Related to Figure 1. (**A**) No correlation was observed between synaptic E_{GABAAR} and the conductance of the light-evoked $GABA_AR$ response (*pooled mean* 10.62 ± 0.6 nS, R^2 = 0.01, p = 0.41, n = 54 neurons from 39 mice). Conductance was calculated from the slope of the $GABA_AR$ current recorded during the voltage ramp. (**B**) No correlation was observed between synaptic E_{GABAAR} and the neuron's series resistance (R_s, *pooled mean* 59.13 ± 1.5 MΩ, R^2 = 0.02, p = 0.33).

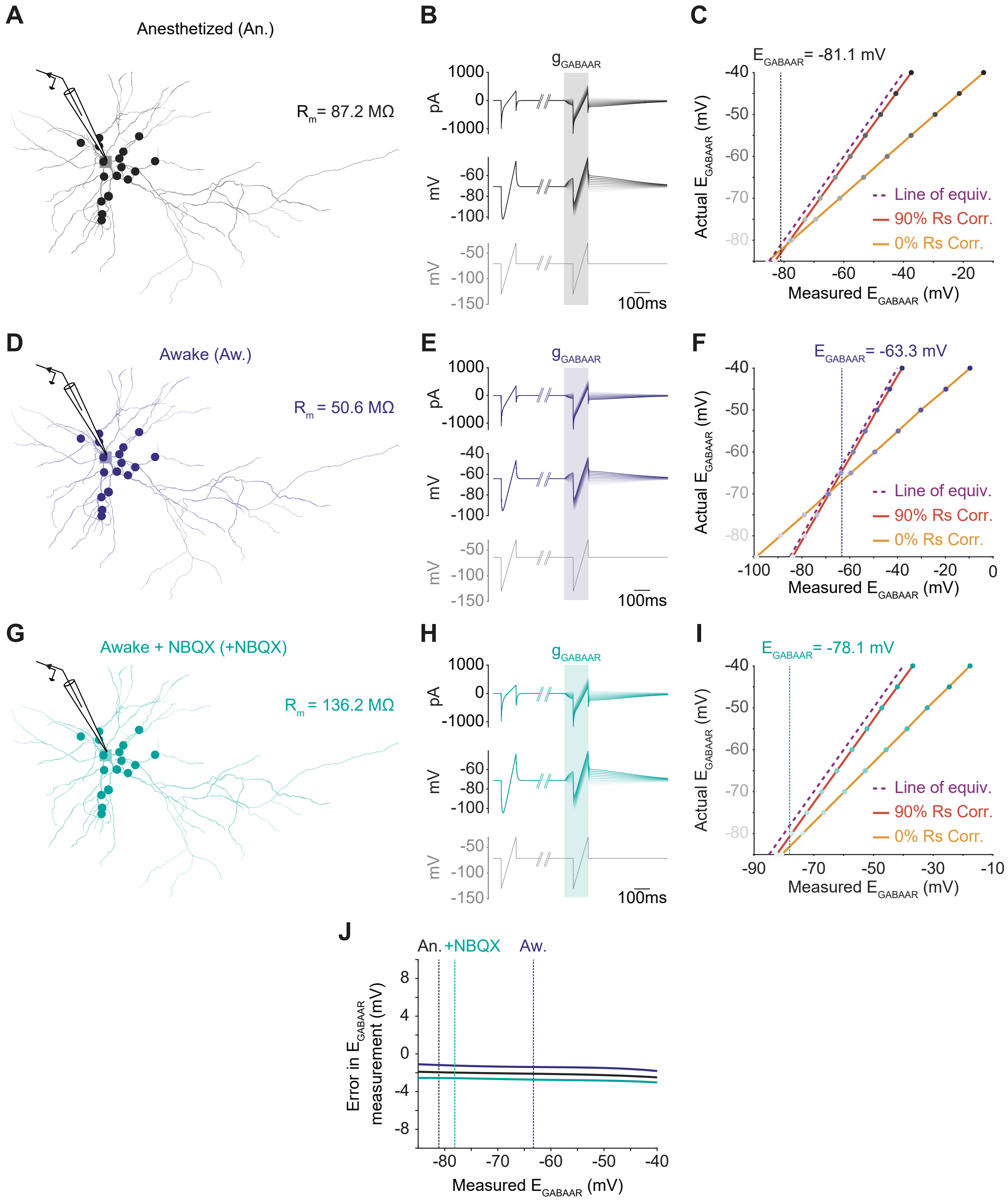

A
Anesthetized (An.)
Rm= 87.2 MΩ
B
gGABAAR
pA
mV
mV
100ms
C
EGABAAR= -81.1 mV
Actual EGABAAR (mV)
Measured EGABAAR (mV)
Line of equiv.
90% Rs Corr.
0% Rs Corr.
D
Awake (Aw.)
Rm = 50.6 MΩ
E
gGABAAR
100ms
F
EGABAAR= -63.3 mV
Line of equiv.
90% Rs Corr.
0% Rs Corr.
G
Awake + NBQX (+NBQX)
Rm = 136.2 MΩ
H
gGABAAR
100ms
I
EGABAAR= -78.1 mV
Line of equiv.
90% Rs Corr.
0% Rs Corr.
J
An.
+NBQX
Aw.
Error in EGABAAR measurement (mV)
Measured EGABAAR (mV)

Supplementary Figure 4: Differences in intrinsic membrane properties have minimal effects upon estimates of synaptic E_{GABAAR}, related to Figures 2 and 3.

To simulate the effect of intrinsic membrane properties on the estimation of synaptic E_{GABAAR} under different experimental conditions, a multi-compartment model of a L2/3 pyramidal neuron from mouse V1 was constructed using NEURON. (**A**) Morphological reconstruction of the L2/3 pyramidal neuron, with the location of $GABA_AR$-mediated synaptic inputs indicated (filled circles). To recapitulate the anesthetized recording conditions (An., black), the membrane resistance (R_m) in the model neuron was set to the mean experimentally observed value in our recordings from anesthetized cortex. (**B**) Voltage ramp protocols that matched the experimental protocols were applied to the model neuron and used to generate measurements of synaptic E_{GABAAR}, which could be compared to the actual E_{GABAAR} that had been preconfigured in the simulated neuron. The different line intensities indicate simulations using different preconfigured E_{GABAAR} values, ranging from -85 mV (lightest line) to -40 mV (darkest line) in 5 mV increments. (**C**) Plot showing the relationship between the actual E_{GABAAR} and measured E_{GABAAR} in the anesthetized model neuron. The line of equivalence (purple) reflects where the actual and measured E_{GABAAR} values are equal. The relationship between the actual and measured E_{GABAAR} values was determined with zero series resistance correction ('0% R_s corr', orange) and with 90% series resistance correction, which corresponds to experimental conditions ('90% R_s Corr.', red). The vertical dashed line indicates the experimentally observed E_{GABAAR} in the anesthetized state. (**D**) As in 'A', except that to recapitulate the awake state (Aw., blue) the membrane resistance and RMP in the model neuron was set to the mean experimentally observed values in our recordings from awake cortex. (**E**) As in 'B', showing simulations of the awake state. (**F**) As in 'C', with the vertical dashed line indicating the experimentally observed E_{GABAAR} in the awake state. (**G**) As in 'A', except that to recapitulate the awake state under conditions of reduced local network activity (+NBQX, turquoise) the membrane resistance and RMP in the model neuron was set to the mean experimentally observed values in our recordings from awake cortex plus NBQX. (**H**) As in 'B', showing simulations of the awake cortex plus NBQX state. (**I**) As in 'C', with the vertical dashed line indicating the experimentally observed E_{GABAAR} in the awake cortex plus NBQX state. (**J**) The estimated error in E_{GABAAR} measurements (Actual E_{GABAAR} – Measured E_{GABAAR}) plotted for each of the three cortical states (using the 90% R_s corrected data). The experimentally measured E_{GABAAR} for each state is indicated with the vertical dashed lines. Across the three states, the estimated error for measuring synaptic E_{GABAAR} was 1-3 mV.

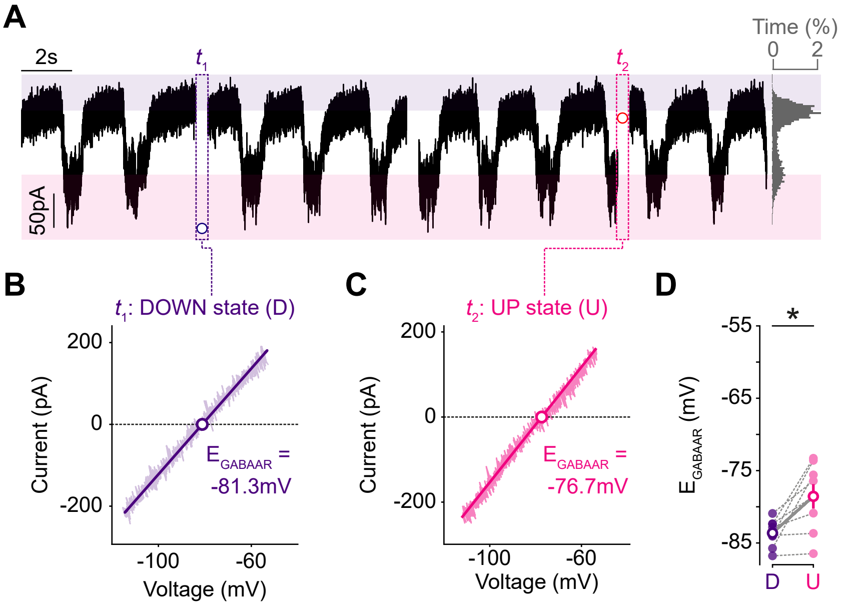

Supplementary Figure 5: Transient fluctuations in E_{GABAAR} are observed across UP and DOWN states in anesthetized cortex, related to Figure 2.

(**A**) More detailed analysis was performed on a subset of anesthetized recordings that exhibited UP and DOWN states. Trace shows a voltage-clamp gramicidin recording from a L2/3 cortical pyramidal neuron in a urethane-anesthetized mouse, showing fluctuations between inward and outward holding currents, corresponding to cortical UP and DOWN states, respectively. Previous work has established that UP states represent periods of high glutamatergic and GABAergic synaptic activity and more depolarized V_m, compared to DOWN states.[2] (**B**) Current-voltage (IV) plot generated from ramp protocol performed during a DOWN state and showing the measured E_{GABAAR}. (**C**) IV plot showing E_{GABAAR} sampled during an UP state. (**D**) Population data showing difference in E_{GABAAR} between cortical DOWN and UP states (DOWN: -83.63 ± 0.68 mV vs. UP: -78.56 ± 1.68 mV, n = 8 cells from 5 mice, p = 0.01, *paired t-test*). This modest synaptic E_{GABAAR} change is consistent with the idea that periods of increased synaptic activity and depolarized V_m favor depolarized synaptic E_{GABAAR} due to an increased Cl^- load.[3,4] *, $p < 0.05$.

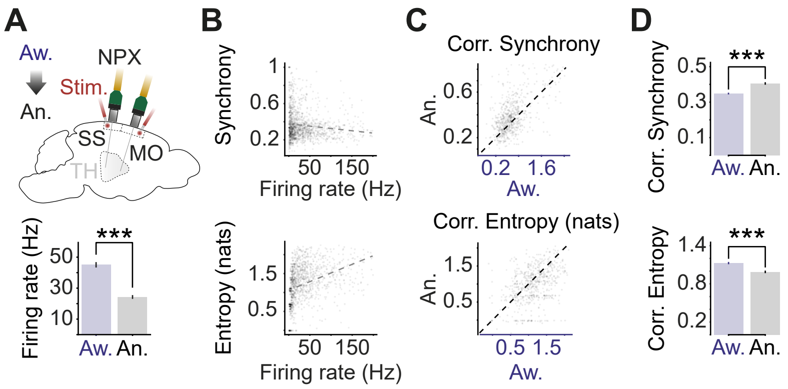

Supplementary Figure 6: Differences in spike rate do not account for differences in synchrony and entropy between the awake and anesthetized cortex, related to Figure 4.

(**A**) Schematic (top) of Neuropixels (NPX) recordings paired with electrical stimulation (Stim.) in somatosensory cortex (SS) and motor cortex (MO) made in the awake (Aw.) and anesthetized (An.) state, from the same mouse. Probes extended from cortex through to thalamus (TH). The firing rate of cortical neurons (bottom; n = 662 neurons from 16 mice) during the awake state was higher than in the anesthetized state (Aw.: 45.1 ± 1.7 Hz vs. An.: 24.3 ± 1.2 Hz, $p < 0.001$, *paired t-test*). (**B**) Scatter plots showing weak relationships between neuron firing rate and synchrony (top; dashed line indicates linear interpolation, $R^2 = 0.02$, $p < 0.001$, *Pearson correlation*) and between firing rate and entropy of the peri-stimulus histogram (bottom; $R^2 = 0.14$, $p < 0.001$, *Pearson correlation*). (**C**) Scatter plots showing synchrony (top) and entropy (bottom) between brain states after correcting for differences in firing rate. (**D**) Population data showing that significant differences in synchrony (Aw.: 0.348 ± 0.005 vs. An.: 0.405 ± 0.007, p <0.001, *paired t-test*) and entropy (Aw.: 1.127 ± 0.017 nats vs. An.: 0.988 ± 0.019 nats, p <0.001, *paired t-test*) remain after correcting for state-dependent differences in firing rate.

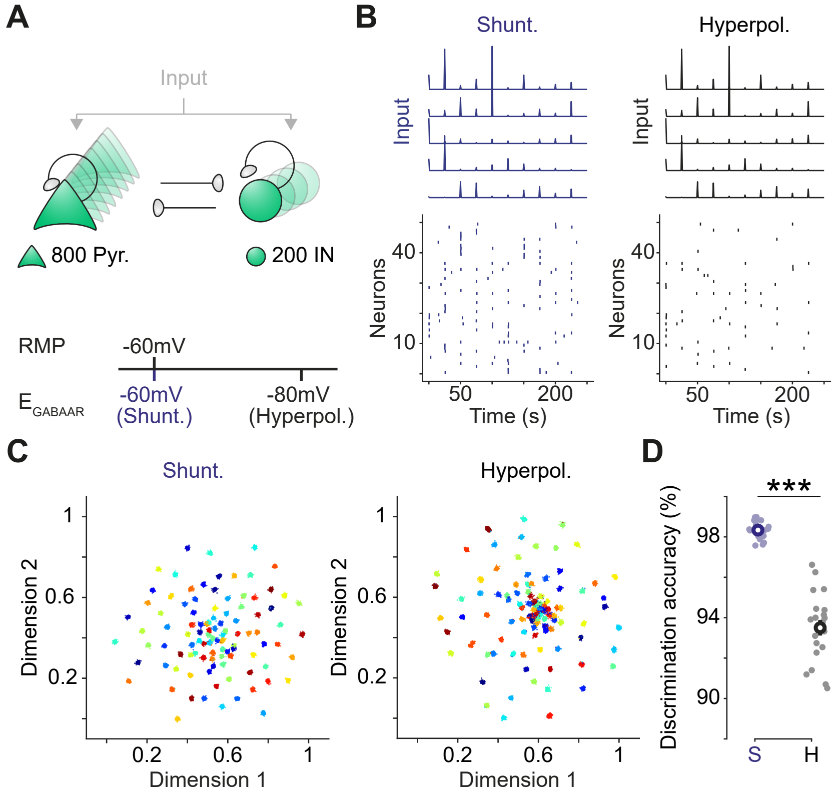

Supplementary Figure 7: Shunting inhibition improves the discriminability of input patterns in neuronal networks, related to Figure 4.

(**A**) Schematic of network model consisting of interconnected excitatory pyramidal neurons (Pyr.) and inhibitory interneurons (IN). E_{GABAAR} in the pyramidal neurons was adjusted relative to the resting membrane potential (RMP) to create two different conditions: a shunting (Shunt.) and a hyperpolarizing (Hyperpol.) E_{GABAAR} condition. For the stimulus inputs (Input), we generated 100 distinct input patterns, each presented 100 times in each condition (shunting or hyperpolarizing E_{GABAAR}). A pattern involved simultaneously delivering a brief current pulse to each neuron, of variable amplitude. (**B**) Example inputs delivered to 5 pyramidal neurons in the model (top). Example raster plots (bottom) show evoked spikes in 50 randomly chosen excitatory neurons in the shunting E_{GABAAR} (left) and hyperpolarizing E_{GABAAR} (right) conditions. (**C**) UMAP projections of the neuronal responses to different input patterns. Each color corresponds to a different input pattern and each dot corresponds to a repeat of that input pattern. In both conditions, responses to the same input pattern form tight clusters. However, shunting E_{GABAAR} results in improved separability of the responses to the different input patterns. (**D**) Discrimination accuracy of k-nearest neighbor classifier trained and tested on input-evoked firing patterns in excitatory neurons (Shunt.: 98.3 ± 0.1 % vs. Hyperpol.: 93.5 ± 0.4 %, n = 20 simulations; $p < 0.001$, *paired t-test*). ***, $p < 0.001$.

Supplementary References:

1. Davies, C.H., Davies, S.N., and Collingridge, G.L. (1990). Paired-pulse depression of monosynaptic GABA-mediated inhibitory postsynaptic responses in rat hippocampus. The Journal of Physiology *424*, 513–531. 10.1113/jphysiol.1990.sp018080.

2. Haider, B., Duque, A., Hasenstaub, A.R., and McCormick, D.A. (2006). Neocortical Network Activity In Vivo Is Generated through a Dynamic Balance of Excitation and Inhibition. Journal of Neuroscience *26*, 4535–4545. 10.1523/JNEUROSCI.5297-05.2006.

3. Doyon, N., Vinay, L., Prescott, S.A., and De Koninck, Y. (2016). Chloride Regulation: A Dynamic Equilibrium Crucial for Synaptic Inhibition. Neuron *89*, 1157–1172. 10.1016/j.neuron.2016.02.030.

4. Staley, K., Soldo, B., and Proctor, W. (1995). Ionic mechanisms of neuronal excitation by inhibitory GABAA receptors. Science *269*, 977–981. 10.1126/science.7638623.
